# The impact of microstructure and extracellular matrix suspension on the proliferation of bone marrow-derived mesenchymal stem cells for osteochondral defect repair

**DOI:** 10.1093/rb/rbaf109

**Published:** 2025-11-12

**Authors:** Elena Stocco, Marta Confalonieri, Silvia Barbon, Carolina Frison, Laura Acquasaliente, Riccardo Boscolo-Pecchie, Valentina Toro Marin, Martina Contran, Rafael Boscolo-Berto, Paola Brun, Silvia Todros, Piero G Pavan, Raffaele De Caro, Veronica Macchi, Andrea Porzionato

**Affiliations:** Section of Human Anatomy, Department of Neuroscience, University of Padova, Padua, 35121, Italy; Department of Women’s and Children’s Health, University of Padova, Padua, 35128, Italy; Department of Surgery, Oncology and Gastroenterology, University of Padova, Padua, 35128, Italy; Foundation for Biology and Regenerative Medicine, Tissue Engineering and Signaling-TES Onlus, Padua, 35121, Italy; Section of Human Anatomy, Department of Neuroscience, University of Padova, Padua, 35121, Italy; Department of Industrial Engineering, University of Padova, Padua, 35131, Italy; Section of Human Anatomy, Department of Neuroscience, University of Padova, Padua, 35121, Italy; Foundation for Biology and Regenerative Medicine, Tissue Engineering and Signaling-TES Onlus, Padua, 35121, Italy; Section of Microbiology, Department of Molecular Medicine, University of Padova, Padua, 35121, Italy; Department of Pharmaceutical and Pharmacological Sciences, University of Padova, Padua, 35131, Italy; Section of Human Anatomy, Department of Neuroscience, University of Padova, Padua, 35121, Italy; Section of Human Anatomy, Department of Neuroscience, University of Padova, Padua, 35121, Italy; Section of Human Anatomy, Department of Neuroscience, University of Padova, Padua, 35121, Italy; Section of Human Anatomy, Department of Neuroscience, University of Padova, Padua, 35121, Italy; Section of Microbiology, Department of Molecular Medicine, University of Padova, Padua, 35121, Italy; Department of Industrial Engineering, University of Padova, Padua, 35131, Italy; Department of Industrial Engineering, University of Padova, Padua, 35131, Italy; Section of Human Anatomy, Department of Neuroscience, University of Padova, Padua, 35121, Italy; National Reference Center for the Conservation and Use of the Bodies of the Deceased, University of Padova, Padua, 35121, Italy; Section of Human Anatomy, Department of Neuroscience, University of Padova, Padua, 35121, Italy; National Reference Center for the Conservation and Use of the Bodies of the Deceased, University of Padova, Padua, 35121, Italy; Unit of Clinical Anatomy, Department of General Surgery, University-Hospital of Padova, Padua, 35121, Italy; Section of Human Anatomy, Department of Neuroscience, University of Padova, Padua, 35121, Italy; National Reference Center for the Conservation and Use of the Bodies of the Deceased, University of Padova, Padua, 35121, Italy; Unit of Clinical Anatomy, Department of General Surgery, University-Hospital of Padova, Padua, 35121, Italy

**Keywords:** oxidized polyvinyl alcohol, porosity, decellularized cartilage, composite porous scaffolds, 3D-printing

## Abstract

Osteochondral defects are a challenge in orthopaedic surgery due to the complexity and function of cartilage. Within this scenario, this study aimed to develop/characterize bioactive porous supports based on oxidized polyvinyl alcohol (OxPVA), with/without human cartilage-derived decellularized ECM (dECM), as platforms for HM1-SV40 cell adhesion and proliferation. OxPVA scaffolds were fabricated using a particle-leaching technique (gelatin concentrations: 10%, 15% and 25% w/w); Scanning Electron Microscopy (SEM) was used to examine the ultrastructure, and a morphometric study assessed pores number, size and porosity percentage. Fluorescence Recovery after Photobleaching (FRAP) was used to evaluate the interconnectivity of the scaffold pores. To enhance the bioactivity of OxPVA, dECM (25% w/w) was incorporated into the scaffolds; thus, the expression of genes related to collagen synthesis and cartilage differentiation/remodelling in seeded HM1-SV40 cells was analyzed by quantitative PCR; relative protein expression levels of SOX9, ACAN and COMP were also assessed. Composite scaffolds biocompatibility was proved by subcutaneous implantation in Sprague–Dawley. As for bone, 3D-printed polylactic acid (PLA)-based scaffolds with varying geometries (67%, 53% and 40% porosity; 600–1400 µm pores size) were fabricated and tested *in vitro*. Lower gelatin concentrations led to numerous superficial pores, whereas higher concentrations produced larger, coalescing ones. HM1-SV40 cells showed better adhesion to scaffolds prepared with 25% gelatin. The OxPVA+dECM scaffolds exhibited a homogeneous matrix distribution, further promoting cell interaction, with a reduction in mean pore size versus matrix-free scaffolds. Moreover, OxPVA supports prepared with 25% gelatin + dECM provided a favorable environment supporting chondrogenic differentiation and cartilage matrix deposition. No inflammatory response to the implants was observed *in vivo*. All PLA supports showed good cell viability; SEM higlighted full-thickness HM1-SV40 cell distribution on and within PLA scaffolds, indicating complete colonization. Further studies are needed to evaluate stem cell differentiation, but bioactive OxPVA and 3D-printed PLA scaffolds show potential for osteochondral regeneration.

## Graphical abstract

**Figure:**
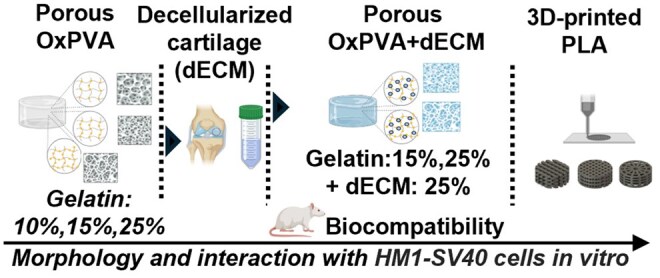

## Introduction

Articular cartilage distinguishes for its avascular, aneural and alymphatic nature, characterized by a limited ability to heal spontaneously in case of damage [1, 2]. It descends that cartilage lesions, due to trauma or degenerative pathology, are associated with joint pain, functional impairment and often progress to osteoarthritis (OA) development, whose management certainly represents a significant clinical challenge [3–5]. To date, beyond conservative drug therapies [6], several approaches have been attempted in clinical practice to promote focal and full-thickness cartilage defects regeneration, including microfracture (marrow stimulation), autologous chondrocyte implantation (ACI), matrix assisted chondrocytes implantation (MACI) and osteochondral autografts/allografts [4, 7, 8]. However, despite they are broadly adopted, notable limitations and drawbacks still exist leading to unsatisfactory outcomes. Typically, these treatments fail in regenerating a native-like cartilage, carry the risk of donor site morbidity and require long and complex surgery [1, 7, 9, 10]. It can be stated that chondral and osteochondral defects, together with OA-associated lesions, currently remain an open challenge in orthopedics and tissue engineering of the musculoskeletal system [11].

To engineer a tissue displaying high water content, like articular cartilage (it is made of up to 80% water), hydrogels were identified as ideal biomaterials. Together with natural hydrogels (e.g. protein-based hydrogels: collagen, elastin, fibrin, gelatin and silk fibroin; polysaccaride-based hydrogels: glycosaminoglycans, alginate, chitosan and agarose; decellularized hydrogels) the use of biodegradable synthetic materials (e.g. poly(ethylene glycol), PEG; poly(N-isopropylacrylamide, pNiPAAm; poly(vinyl alcohol), PVA) is also reported [12], being able to recapitulate the cartilage solid/liquid ratio [11]. Moreover, their intrinsic characteristics (chemistry, crosslinking density, degradation rate, mechanical properties and biochemical factors release kinetics) together with ultrastructure can be tuned, to mimic natural extracellular matrix (ECM) architecture and function, thus, suiting the desired biomedical application [1, 8].

Due to submicron- or nano-sized gel networks, hydrogels may obstacle cell growth, matrix deposition and tissue formation as consequence of restricted oxygen and nutrients supply. Thus, in recent years, the introduction of macropores within the hydrogels have shown great potential to overcome this problem [13]. Several methods have been adopted to control hydrogel porosity, these include solvent casting, freeze-drying, gas foaming, 3D printing, micropatterning and porogen leaching [13–15]. Porogen leaching is recognized among the first processing approaches to make porous scaffolds; once particles are dispersed within a hydrogel, the system is gelled/fixed; consequently, porogen removal results in a porous morphology of the support [16]. In this study, the polymer *oxidized polyvinyl alcohol* (OxPVA) [17] was specifically considered for fabrication of porous scaffolds aiming at cartilage tissue engineering purposes. In fact, OxPVA-derived hydrogels, despite several appealing characteristics of tunability and broad use for tissue engineering purposes [17–27], lack in promoting cell adhesion and proliferation (like the native counterpart PVA), possibly as consequence of a too high hydrophilicity [7, 28]. It descends that strategies are required to overcome this limit, while continuing in taking advantages from derived-hydrogels potentials. Porous scaffolds exert a fundamental role as a temporary support for cells [29]; here, gelatin was used as a leachable porogen; specifically, different gelatin percentages were used to investigate the tunability of pore size and porosity of OxPVA hydrogels. Following preliminary *in vitro* assays, the further contribution of ECM derivatives was focused, fabricating porous OxPVA scaffolds + articular cartilage ECM. It is well established that ECM-derived scaffolds are attractive for regeneration/replacement of damaged tissue and organs [30, 31]; thus, derived lysates can be used to introduce different biological cues within scaffolds, like proteins, glycosaminoglycans and cytokines, favoring cell attachment, proliferation and differentiation [7, 21, 32]. For the first time, decellularized human cartilage was mixed, once homogenized, with OxPVA for a whole hydrogel bioactivation; composite scaffold biocompatibility was considered through subcutaneous implantation in Sprague–Dawley rats, to assess evidence of safety.

Recent advancements in bone tissue engineering include three-dimensional (3D) printing technology, which is used to build-up biodegradable porous scaffolds that mimic the inner morphology/microstructure of human bone, thus, serving as excellent templates for the ingrowth of bone [33, 34]. Fused deposition modeling (FDM) technology is broadly adopted allowing to print high molecular-weight polymers according to a certain configuration and pore structure [33]. Biopolymers that are mainly used in 3D printing include polycaprolactone (PCL), polylactic-co-glycolic acid (PLGA) and polylactide acid (PLA) which have proved their suitability for bone defects treatment also by virtue of ease processing and generation of nontoxic, resorbable degradable products [35]. Here, 3D printed PLA scaffolds were realized with different geometries, and thus, assayed for specific interactions with mesenchymal stem cells in the perspective of complex, bilayered composite scaffolds fabrication (porous composite OxPVA + 3D printed PLA) aiming at osteochondral repair.

## Materials and methods

### Preparation of oxidized polyvinyl alcohol hydrogel

The oxidized polyvinyl alcohol (oxidation degree: 1%; from here on, OxPVA) solution was prepared in accordance with Stocco *et al*. [17]. Briefly, a weighted amount of PVA powder [molecular weight (Mw) 146 000–186 000 Da, 99+% hydrolysed] (Sigma-Aldrich, St Louis, MO, USA) was suspended in MilliQ water. The system was then heated to 100°C under continuous stirring for 48 h to ensure complete dissolution of the polymer. Subsequently, the oxidative solution, made of potassium permanganate (KMnO_4_) in dilute perchloric acid (HClO_4_), was added. Typically, 1 g PVA was dissolved in 20 ml of MilliQ water, followed by the addition of 5 ml of an aqueous KMnO_4_ solution (2.9 mg/ml) + 0.3 ml of 70% HClO_4_. The reaction was carried out at 30°C for 1 h. Following PVA oxidation, the polymer was extensively dialyzed through 8000 Da cut-off membranes (Sigma-Aldrich, Steinheim, Germany) and frozen at −20°C overnight before lyophilization (Speed Vac Concentrator Savant, Instruments Inc., Farmingdale, NJ, USA). Polymer recovery (16% w/v) was obtained by suspending a weighted amount of OxPVA in dH_2_O (deionized water) and heating the system in a laboratory stove to 95°C, until complete OxPVA dissolution.

### Porous OxPVA scaffolds fabrication and characterization

#### Porous scaffolds setup by particulate-leaching technique

Porous OxPVA scaffolds were obtained by particulate-leaching method. Specifically, OxPVA hydrogel solution, at 37°C, was poured onto a glass plate and mechanically incorporated with gelatin microspheres (gelatin from bovine skin, Sigma Aldrich) using a laboratory spatula. To ensure proper dispersion of the gelatin powder within the OxPVA solution, the glass plate was positioned on a laboratory hot plate set to 40°C, to maintain an adequate temperature for the OxPVA. Three different percentages of gelatin were used: 10%, 15% and 25% (w/w to OxPVA), respectively; the gelatin powder was gradually added in small amounts under continuous stirring, to prevent the formation of lumps. Hence, the mixtures were transferred within customized PLA moulds (diameter (Ø) = 0.7 cm; height (*h*) = 0.2 cm) that were previously 3D-printed using a Ultimaker 2+ Connect 3D printer (Ultimaker, Utrecht, Netherlands). The systems were physically cross-linked through six freeze-thawing cycles; each cycle consisted in a freezing phase at −80°C for 2 h followed by a thawing phase at room temperature (RT) for 30 min. Gelatin was then removed from the cross-linked scaffolds by dissolution in dH_2_O, under stirring, at 40°C. Subsequently, the scaffolds were gently patted dry to remove excess water and stored at−20°C until use.

The porous scaffolds were then evaluated/compared for ultrastructure, and specific bioactivity.

In parallel, smooth OxPVA scaffolds were also fabricated as control group. Their fabrication followed the same protocol, excluding gelatin powder incorporation.

#### Ultrastructure evaluation by scanning electron microscopy and porosity measurement

Ultrastructure of OxPVA porous scaffolds was investigated by SEM. Once the samples were fixed with 2.5% (v/v) glutaraldehyde in 0.2 M PBS for at least 48 h, they were washed with phosphate buffer and dehydrated by immersion in an ascending grade series of ethanol (Arco Scientifica and S.r.l., Padua, Italy). After critical point drying and gold sputtering, micrographs were acquired by a JSM-6490 Scanning Electron Microscope (Jeol USA, Peabody, MA, USA).

Five images at 300x magnification were taken from each sample and these were then processed with the ImageJ software (ImageJ 1.54f, Rasband W. and contributors, National Institutes of Health, USA) to estimate the overall porosity of the samples. Specifically, the total pores number, the pores average size and the % area occupied by the pores were estimated.

#### Mechanical characterization of porous scaffolds

The compressive behavior of porous scaffolds (i.e. OxPVA, OxPVA + 10%P, OxPVA + 15%P, OxPVA + 25%P), was evaluated using a Bose ElectroForce^®^ Planar Biaxial Test Bench (TA Instruments, New Castle, DE, USA) equipped with a 22 N load cell. Five samples per each experimental group were tested fully submerged in PBS to mimic physiological conditions. A flat compressive plate was brought into contact with the specimen surface (considering a compressive force threshold equal to −0.005 N), and then, moved forward up to a compression of 20% of the initial sample height. This compressive level is consistent with the maximum physiological strain of human cartilage [36–38]. This loading condition was then maintained for 3600 s to assess consolidation. Nominal stress was calculated as instantaneous force divided by initial surface area, and nominal strain as displacement relative to the initial sample thickness. Stiffness was expressed as the secant modulus at 20% strain, obtained by dividing nominal stress by the maximum compressive strain. Force relaxation during consolidation was quantified by dividing the instantaneous force by the initial maximum force value.

#### Florescence recovery after photobleaching on porous scaffolds

Porous scaffolds underwent fluorescence recovery after photobleaching (FRAP) experiments. The tests were conducted at RT using a Zeiss LSM800 Airyscan confocal microscope (Carl Zeiss Microscopy GmbH, Jena, Germany) equipped with a 20x objective. Thus, the samples were incubated for 24 h in 1x PBS containing 2 mg/ml green fluorescein isothiocyanate (FITC)-dextran (500 kDa) at RT. A circular region of interest (ROI, 30 μm diameter) was photobleached with a 488 nm argon laser at 100% intensity for 32 s, corresponding to 500 iterations. Image acquisition comprised 45 frames captured at 1 s intervals, including five pre-bleaching images. A second ROI of identical size, positioned in the opposite corner of the frame, served as an unbleached control. For each sample, measurements were taken at five distinct locations. Fluorescence recovery curves were obtained by normalizing the integrated fluorescence intensity of the bleached ROI at each time point to its pre-bleaching value. The half-recovery time, defined as the time necessary to the fluorescence signal to recover to 50% of its pre-bleached intensity, was calculated using a house-developed MATLAB code (MATLAB version: R2024b, Natick, Massachusetts, USA).

#### Evaluation of porous scaffolds cytocompatibility

Scaffolds’ cytocompatibility was evaluated *in vitro* by seeding the porous scaffolds with immortalized human bone marrow mesenchymal stem cells (HM1-SV40) and measuring cells metabolic activity/viability through MTT (3-[4,5-dimethylthiazol-2-yl]-2,5 diphenyl tetrazolium bromide) assay after 7 and 14 days. Smooth OxPVA scaffolds were considered as control group.

##### Cell culture and scaffolds seeding

HM1-SV40 cells were plated into T-25 flasks (Corning, New York, USA) and cultured in a humidified environment at 37°C, 5% CO_2_. Culture medium consisted in Minimum Essential Medium-alpha (MEM-α, Life Technologies, Paisley, United Kingdom) added with 16.5% (v/v) foetal bovine serum (FBS; Sigma Aldrich), 1% (v/v) penicillin/streptomycin (Life Technologies) and 1% (v/v) glutamine; it was refreshed regularly until cells reached 80–90% confluency. Hence, cells were detached by trypsin/EDTA (Sigma Aldrich) solution [0.25% (w/v) trypsin, 0.025% EDTA (w/v) in PBS] according to routine protocols and resuspended with fresh culture medium before scaffolds seeding.

Preliminarily, smooth scaffolds and porous scaffolds, prepared with different amounts of gelatin (10%, 15% and 25% w/w), were sterilized by immersion in sterile PBS containing 2% (v/v) penicillin/streptomycin for 48 h under stirring; after washing in sterile PBS to remove decontaminants residues, supports were exposed under UV light for 20 min each side. Hence, the samples were incubated in culture medium at 37°C, overnight. Scaffolds were then placed in a 48-well plate, seeded with 100 000 HM1-SV40 cells and incubated at 37°C in a 5% CO_2_ humidified environment. The endpoints considered were of 7 and 14 days; culture medium was partly refreshed every 3 days and fully refreshed the day before MTT assay.

##### MTT assay

After 7 and 14 days from seeding, MTT assay was performed. Culture medium was replaced with MEM-α supplemented with 0.5 mg/mL MTT for 4 h; thus, it was removed, and the formazan crystals were solubilized with 2-propanol acid (0.04 M HCl in 2-propanol). Solutions optical density was measured at 570 nm with the Microplate Auto Reader VICTOR3™ (PerkinElmer, Waltham, MA, USA). It was possible to determine the total number of cells/samples through a standard curve correlating number of HM1-SV40 cells and absorbance.

### Articular cartilage decellularization and protocol effectiveness evaluation

Scaffolds displaying the most promising characteristics in terms of interactions with cells were then considered to be combined with decellularized articular cartilage.

### Human articular cartilage sampling

Human articular cartilage was obtained from a not-embalmed cadaver (male, 67-year old) enrolled within the Body Donation Program ‘Donation to Science’ of the University of Padova [31, 39, 40]. The Donor did not have any history of symptomatic OA, or knee comorbidities; signs of cartilage degradation were not detected at dissection. Right and left knees in flexion were dissected; after removal of patellar ligament and patella, the knees were subluxated allowing for full-thickness cartilage excision from the femoral condyles. After tissue removal, cartilage specimens underwent decellularization as described below; in parallel, samples of native cartilage were adequately fixed/processed for subsequent histological/immunohistochemical analysis and ultrastructural characterization studies (used as control).

### Articular cartilage decellularization

Decellularization was performed according to the detergent-enzymatic method by Meezan *et al*. [41], partly modified. Briefly, cartilage tissue was thoroughly washed in PBS supplemented with 2% (v/v) penicillin/streptomycin (Sigma-Aldrich), then, transferred to a Petri dish and finely minced using a scalpel blade. Hence, the fragments were placed in a 50 ml tube and treated with dH_2_O at 4°C under agitation for 72 h; dH_2_O was changed every 2 h. Thus, specimens were soaked in 4% (w/v) sodium deoxycholate (C_24_H_39_NaO_4_, Sigma Aldrich) solution at RT, under agitation, for 4 h and lastly, they were exposed to 2000 Kunitz units of DNAase-I (Deoxyribonuclease I from bovine pancreas, Sigma Aldrich) in a 1 M sodium chloride (NaCl) solution at RT, under agitation for 2 h. This procedure, constituting a single decellularization cycle, was repeated until complete decellularization was verified. The decellularized tissue is referred to as decellularized extracellular matrix (dECM).

All procedures were performed under a laminar flow hood, in sterile conditions and using sterile equipment/materials and solutions.

### 4^’^,6-Diamidino-2-phenylindole nuclear staining

4′,6-Diamidino-2-Phenylindole (DAPI) fluorescent staining allows to evaluate nuclei presence and distribution within samples. Briefly, native and decellularized cartilage fragments were soaked in Optimal Cutting Temperature (OCT) medium and frozen at −80°C prior to be cut in 5 µm thick sections through a cryomicrotome (Leica CM 1850 UV; Leica Microsystems, Wetzlar, Germany). Hence, sections were allowed to dry and immersed in dH_2_O for 15 min prior to be incubated in the dark with DAPI solution for 10 min. Subsequently, sections were drained, washed with dH_2_O and mounted with Mowiol^®^ mounting medium.

Photomicrographs were acquired with a Leica LMD6 connected to a Leica DFC320 high-resolution digital camera and a computer equipped with software for image acquisition (LasX, Leica Microsystems).

### Residual DNA extraction and quantification

Detection of eventual immunogenic material residues in decellularized tissue versus native tissue was performed by DNA quantification. The DNeasy^®^ Blood & Tissue Kit (Qiagen, Düsseldorf, Germany) was used. Briefly, 10 mg of tissue were lysed with Proteinase K (Merck Life Science, Darmstadt, Germany) at 56°C overnight and the lysates were loaded onto the DNeasy Mini spin columns for selective purification of total DNA. Eluted DNA underwent fluorometric quantification by Qubit 4 fluorometer and kit (ThermoFisher Scientific, Waltham, MA, USA). Three replicates/group were analyzed.

### Decellularized cartilage characterization

#### Histological analyses

Histological analyses were performed to characterize the articular cartilage ECM following decellularization. Native and decellularized cartilage samples were fixed in 10% formalin solution for at least 48 h, and paraffin embedded according to routine laboratory protocols. Thus, 5 µm thick sections were dewaxed and rehydrated with a series of ethanol (Arco Scientifica) solutions (99%, 95%, 70%) and dH_2_O. Subsequently, Azan–Mallory staining allowed to evaluate total collagen distribution; Sirius Red staining allowed to distinguish collagen type I/III through visualization under polarized light; Alcian Blue was carried out to assess the glycosaminoglycans (GAGs) presence.

#### Glycosaminoglycans quantification

Sulfated GAGs content in the samples was quantified using the Chondrex Inc. Glycosaminoglycans Assay Kit (Woodinville, WA, Stati Uniti), following the manufacturer’s protocol. To achieve GAGs solubilization, 10 mg of tissue samples were preliminary incubated at 60°C overnight with 1 ml of a papain solution [0.05 M PBS (pH 6.5), 5 mM cysteine, 5 mM EDTA and 125 µg/ml papain]. Hence, the suspension was centrifuged at 10 000 rpm for 5 min and the supernatant was transferred to a 1.5 ml microcentrifuge tube. For the assay, a 5-point standard curve was prepared by serially diluting chondroitin-6-sulfate in PBS, thus, standards, blank and samples were transferred into the wells of a 96-well plate and 1.9 dimethylmethylene blue (DMB) dye solution was added. The plate was immediately read at 530 nm using the VICTOR3™ (PerkinElmer) Microplate auto reader.

#### Ultrastructure evaluation by scanning electron microscopy

Native and decellularized cartilage samples were analyzed through SEM to evaluate cartilage tissue ultrastructure before and after decellularization and consider eventual impact of the treatment over tissue appearance. Tissue samples, once fixed with 2.5% (v/v) glutaraldehyde in 0.2 M PBS for at least 48 h, were processed as previously described.

### Fabrication and characterization of porous scaffolds combined with decellularized cartilage

#### ECM suspension

Decellularized cartilage fragments (1 g) were placed in a plastic tube and soaked with 15 ml of 10% (v/v) acetic acid solution (CH_3_COOH, Carlo Erba, Cornaredo, Italy). Thus, the tissue was homogenized at 0°C for 20 s, 8 times, at intervals of 6 min using a homogenizer (ULTRA-TURRAX^®^, IKA, Staufen, Germany). The suspension was finally centrifuged at 2000 rpm for 5 min and, once discarded the eventual deposits at the tube bottom, it was homogeneously resuspended and stored at −20°C until use.

#### Porous composite OxPVA + dECM scaffolds fabrication and characterization

Porous composite OxPVA + dECM scaffolds were fabricated by incorporating 25% (w/w to OxPVA) of dECM suspension to the OxPVA previously mixed with gelatin powder (OxPVA + 15%P and OxPVA + 25%P). Briefly, the dECM suspension was thawed in a water bath at 37°C. Following the mechanical incorporation of 15%P and 25%P gelatin into OxPVA (as previously described), the dECM suspension was added drop by drop and carefully mixed to ensure uniform distribution.

The resulting OxPVA/gelatin/dECM mixtures were then transferred into PLA molds, and six freeze–thaw cycles (−80°C for 2 h/RT for 30 min) were applied to induce scaffold cross-linking. Afterward, gelatin was removed by dissolution in dH_2_O under stirring at 40°C. The scaffolds were, then, gently patted dry to remove excess water and stored at −20°C until use.

#### Histological characterization

Histological analyses were performed to evaluate the distribution of the dECM within the scaffolds. Frozen porous supports with (+) and without (−) dECM were fixed in 10% (v/v) formaldehyde for at least 48 h. Thus, the samples were processed as described above and 5 µm thick sections were stained with Hematoxylin&Eosin (H&E) and Alcian blue.

#### Ultrastructure analysis by scanning electron microscopy and porosity evaluation

Porous composite OxPVA + dECM scaffolds were analyzed by SEM to evaluate specific ultrastructure after dECM incorporation. The samples were prepared as previously described; thus, five images/specimen at 300x magnification were taken from each sample for the morphometric study.

#### Mechanical characterization of composite scaffolds

The compressive behavior of porous scaffolds, with acellular matrix enrichment (i.e. OxPVA + dECM, OxPVA + 15%P+dECM, OxPVA + 25%P+dECM) was evaluated as previously described for porous scaffolds based on OxPVA only.

#### Florescence recovery after photobleaching on composite scaffolds

The diffusivity properties of porous scaffolds enriched with acellular matrix were evaluated by means of FRAP experiments, following the same protocol previously described.

#### Evaluation of porous composite scaffolds cytocompatibility

Porous composite scaffolds, once decontaminated, were evaluated for cytocompatibility *in vitro* by seeding the scaffolds with 100 000 HM1-SV40 and performing a MTT assay after 7 and 14 days, as previously described.

#### RNA isolation and quantitative real-time PCR

Total RNA was prepared from (100 000 seeded cells) cells at 7 and 14 days of culture using the SV total RNA isolation system (Promega, Milan, Italy). Contaminating DNA was removed by DNase I treatment (Promega). Five µg of total RNA were reverse-transcribed and amplified using iTaq Universal One-Step RT-qPCR Kit (Bio-Rad, Segrate, Italy), containing iScript RNase H, MMLV reverse transcriptase, hot-start iTaq DNA Polymerase and SYBR^®^Green. The primers used in this study were designed using the online Primer 3 software and synthesised by Merck (Milan, Italy). The sequences of the primers are reported in Table 1. Quantitative polymerase chain reaction (qPCR) was performed using the QuantStudio Real-Time PCR Systems (ThermoFisher, Milan, Italy) for 40 cycles at the annealing temperature of 60°C. The expression levels of the target genes were normalised to the endogenous control gene β-*ACTIN* and calculated using the 2^−ΔΔCt^ method.

**Table 1.** Sequence of primers used in the quantitative real-time PCR analysis

Gene	Oligonucleotide (5′–3′)	
	Forward	Reverse
*COL2A1*	TTCAGCTATGGAGATGACAATC	AGAGTCCTAGAGTGACTGAG
*COL9A1*	GTTGCAAACGGTGCACCTAC	GAGAGCTTGAGGTTCTCGGG
*COL10A1*	CACCAGGCATTCCAGGATTCC	AGGTTTGTTGGTCTGATAGCTC
*COMP*	GGGCCCGCAGATGCTTC	GGTTTGTTGGTCTGATAGCTC
*SOX5*	AGCCAGAGTTAGCACAATAGG	CATGATTGCCTTGTATTC
*SOX9*	GAACGCACATCAAGACGGAG	TCTCGTTGATTTCGCTGCTC
*BMP2*	CAGAGACCCACCCCCAGCA	CTGTTTGTGTTTGGCTTGAC
*BMP6*	CTCGGGGTTCATAAGGTGAA	ACAGCATAACATGGGGCTTC
*MMP14*	ATGGCCACCGTTAAGAAGGG	GCGATGACACCAGCATTGTC
*MMP16*	TTCCTACCCACTGCTTGCTG	CAGTGAGGCAAGCCGAAGTA
*P4HA1*	AAATGACGTCTGGGCTCTGG	CTAGCGTGGAAAGTTCGGGT
*Β-ACTIN*	CCAAGGCCAACCGCGAGAAGAT	AGGGTACATGGTGGTGCCGCCA

#### Semiquantitative proteins detection via dot blot

Dot blot analysis enabled the semiquantitative detection of proteins encoded by SOX9, ACAN and COMP, secreted by HM1-SV40 cells (100 000 seeded cells) following 14 days of culture on OxPVA-based scaffolds. For each gene/group, 1 µg of total protein was spotted onto a nitrocellulose membrane (0.45 µm pore size, GE Healthcare). Thereafter, the membrane was air-dried for 30 min at RT, then blocked in 5% nonfat dry milk in TBS-T (Tris-buffered saline with 0.1% Tween-20) for 1 h at RT under gentle agitation. After blocking, membranes were incubated overnight at 4°C with primary antibodies diluted in blocking buffer. Specifically, the following antibodies were used: anti-SOX9 (SOX9 Rabbit mAb, ABclonal Biotechnology; diluted 1:1000), anti-ACAN (Aggrecan Rabbit pAb, ABclonal Biotechnology, Wuhan, China; diluted 1:1000) and anti-COMP (COMP Rabbit pAb, ABclonal Biotechnology; diluted 1:1000). Following incubation, membranes were carefully washed for 3 times (10 min each) with TBS-T and then incubated for 1 h at RT with the HRP-conjugated secondary antibody (1:2000, SoutherBiotech). Proteins were visualized using an enhanced chemiluminescence substrate (Cytiva) and detected using a Azure400 imaging system (AzureBiosystem). Spot intensity semi-quantification was performed using ImageJ software, and values were normalized to a reference control (HM1-SV40 subconfluent cells).

#### Biocompatibility in vivo

*In vivo* biocompatibility of porous composite scaffolds was evaluated by subcutaneous implantation in Sprague–Dawley rats. Animal surgery and husbandry was approved by the Ethical Committee of the University of Padova and by the Italian Department of Health (Authorization n.782/2021-PR; date of release: October 15, 2021).

##### Surgery

Eight Sprague–Dawley rats (two animals/group) were anesthetized with an isoflurane/oxygen gas mixture; thus, the dorsal area was shaved, disinfected with Betadine^®^ (Bayer, Leverkusen, Germany) and a median 10 mm dorsal incision allowed to create a subcutaneous pouch. Scaffolds were fixed to the latissimus dorsi muscle by using Tycron 4/0 sutures. Finally, absorbable Novosyn 4/0 sutures were used to stich the skin. After surgery, enrofloxacin (Baytril^®^ 5 mg/kg) and tramadol (Altadol^®^ 3–4 mg/kg) therapy was administered for 3 days.

Fourteen days after implantation, rats were euthanized with carbon dioxide; thus, the implants and the surrounding tissues were excised and properly fixed for histological/immunohistochemical analyses, and SEM investigation.

##### Explants histological and immunohistochemical characterization

Formalin-fixed, paraffin-embedded sections of explants were prepared as previously described. H&E and Azan–Mallory staining were performed. In parallel, lympho-monocytic fraction was immunolocalized by using the following primary antibodies: anti-CD3 (polyclonal rabbit anti-CD3, A0452, Dako, Italy; 1:500) and anti-F4/80 (polyclonal rabbit anti-F4/80 (M-17)-R, sc-26643-R, Santa Cruz Biotechnology, USA; 1:1000) to label lymphocytes and monocytes/macrophages, respectively. Antigen retrieval was performed in 10 mM sodium citrate buffer, pH 6.0 at 90°C for 10 min. The sections were then incubated in peroxidase blocking solution (Dako) to eliminate unspecific reactivity and then incubated for 45 min with the above primary antibodies. Hence, sections were incubated for 45 min with anti-rabbit secondary antibodies and reactivity was revealed by incubation with Horseradish peroxidase-linked polymer (Dako) for 20 min and developed in 3,3′-diaminobenzidine for 3 min. Lastly, sections were counterstained with hematoxylin.

##### Ultrastructure analysis by scanning electron microscopy

Ultrastructure characterization of explanted scaffolds was performed by SEM, as previously described.

### PLA scaffolds fabrication and characterization

PLA scaffolds were fabricated using computer-aided design (CAD) software and 3D printing. Three different geometries were investigated and compared to analyze their ultrastructure and specific interaction with cells.

### Scaffolds design by computer-aided and fabrication by 3D printing

Structures were designed using the Fusion 360 CAD software (Autodesk, San Rafael, CA, USA). All the scaffolds were designed with a diameter of 8.5 mm and a height of 4.0 mm to allow them to be placed inside the wells of a 48-well plate. Parameters for 3D printing were set with the software of the printer (UltiMaker Cura v. 5.3.1; nozzle diameter, 0.4 mm; layer height, 0.04 mm). Scaffolds were printed in PLA using the FDM 3D printer Ultimaker 2+ Connect (Ultimaker), which build the structures layer by layer by melting and extruding the material in the form of filaments. At last, 3D-printed scaffolds were refined using a commercial milling machine. Printed scaffolds characteristics are reported in Table 2.

**Table 2.** PLA scaffolds geometrical parameters

Scaffold		Pore size, µm		Total porosity, %	Fiber width, mm
Geometry 1	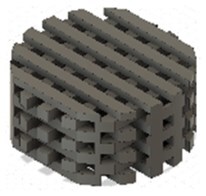	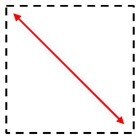	990	67	0.65
Geometry 2	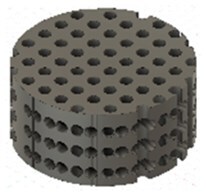	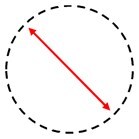	600	53	0.50
Geometry 3	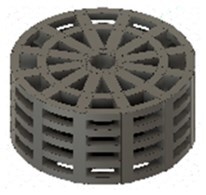	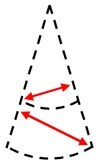	700–1400	40	0.60

### Scaffolds ultrastructure evaluation by scanning electron microscopy

Plain 3D-printed PLA scaffolds were directly subjected to gold sputtering and then analyzed with the JEOL JSM-6490 Scanning Electron Microscope to evaluate their ultrastructure.

### Evaluation of scaffolds-cell interaction

Cells interaction with different PLA geometries was assayed *in vitro* by seeding the scaffolds with 100 000 HM1-SV40 cells and performing a MTT assay; end-points: 7 and 14 days.

### Statistical analysis

All experimental data were expressed as mean ± standard deviation (SD). Experimental data are expressed as mean ± standard deviation (SD). Statistical analysis was performed using GraphPad Prism version 8.4.2 for Windows (GraphPad Software, San Diego, CA, USA). For comparisons between two groups, an unpaired *t*-test was used; for multiple group comparisons, one-way ANOVA followed by Tukey’s *post hoc* test was applied. When data did not meet parametric assumptions, the Kruskal–Wallis test followed by Tukey’s *post hoc* test was used. Statistical significance was set at *P* < 0.05.

For cell proliferation assays, gene expression analyses and semi-quantification of secreted proteins comparisons were performed separately at each time point (7 and 14 days) to evaluate treatment-specific effects. Scaffold porosity was quantified from SEM images by analyzing morphometric parameters (including total pore number, average pore size and pore-covered area). Five images per group (acquired at 300× magnification and processed in ImageJ) were considered as independent technical replicates (*n *= 5 per group). Glycosaminoglycan (GAG) and DNA content were quantified with at least three replicates in two experimental groups (NativeC vs DecellC). Mechanical testing was performed on five samples per group, and differences in the secant modulus were assessed. For porous scaffolds, four experimental groups were compared, including conditions with and without dECM. In FRAP analyses, τ1/2 values were compared across groups, with five ROIs analyzed per sample (*n *= 1 per group).

## Results

### Porous scaffolds fabrication and ultrastructure comparison

While incorporating gelatin powder (10%, 15%, 25% w/w) within OxPVA solution, the polymer progressively assumed a paste-like appearance. Hence, it was possible to model it within the molds and proceed with freeze-thawing. Following crosslinking, porogen removal took about 45–60 min for all the three conditions.

In comparison to the control group (smooth, cross-linked OxPVA scaffolds), the porous OxPVA scaffolds displayed a macroscopically rougher surface with visible pores. As expected, scaffolds made with higher gelatin percentages appeared rougher than those with lower gelatin percentages (roughness: 25%*P* > 15%*P* > 10%P).

SEM micrographs were performed to characterize the superficial and transversal ultrastructure of the scaffolds. This analysis confirmed a smooth and regular structure for the control group; whereas, a different morphology, characterized by a rough, porous structure was typical of the supports prepared with different porogen amounts. At higher magnification, scaffolds prepared with lower gelatin percentage (10%P) showed a more homogenous distribution of pores compared to scaffolds fabricated with a higher gelatin percentage (Figure 1A).

**Figure 1.**
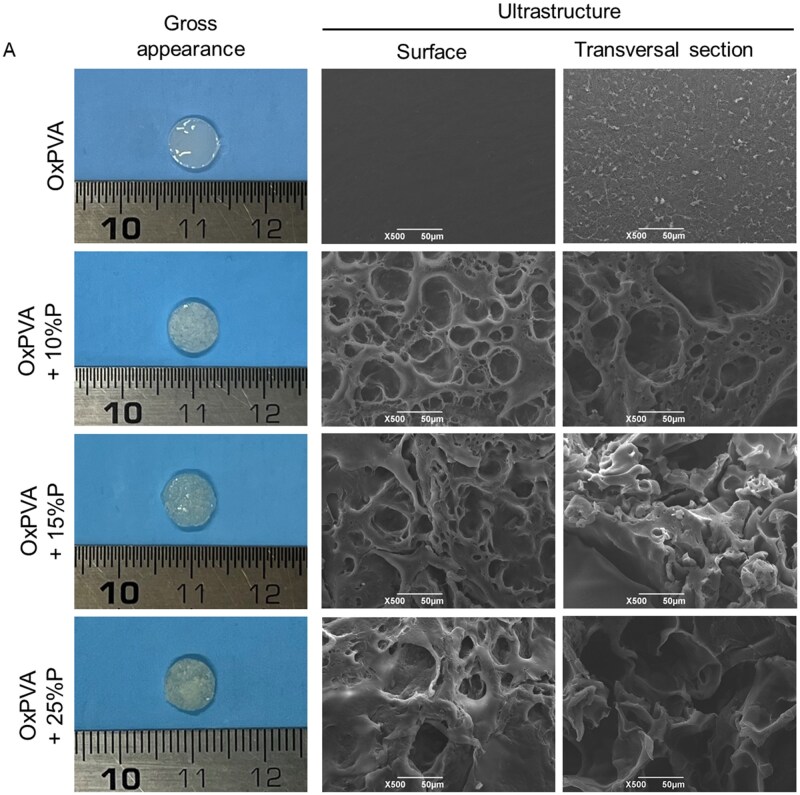
Porous scaffolds comparison. (**A**) Gross appearance and ultrastructure by SEM of OxPVA-based scaffolds. Scaffolds were fabricated without (OxPVA) or with different percentages of porogen (P): OxPVA + 10%P, OxPVA + 15%P and OxPVA + 25%P; overall appearance of surface and transversal sections was also shown (scale bar: 50 µm). (**B**) Porosity assessment and morphometric study. Analyzing SEM photomicrographs, surface and transversal sections of the scaffolds were compared for pores count (B, a, d), pores average size (B, b, e) and pores % area (B, c, f). (*P *<* 0.05; **P *<* 0.01; ***P *<* 0.001; ****P *<* 0.0001).

**Figure 1.**
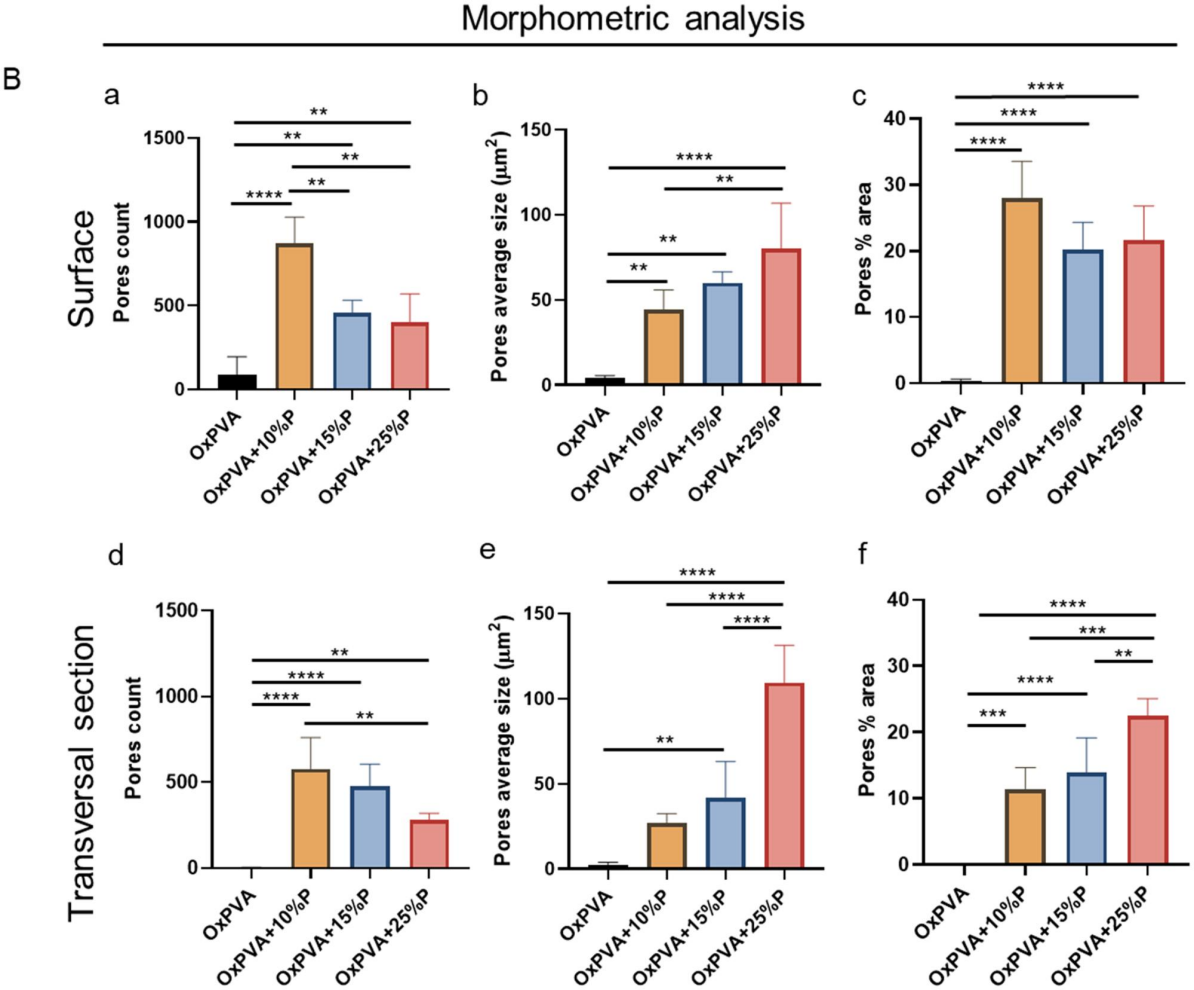
(Continued)

Considering the importance of pores for scaffolds bioactivity, the surface and transversal sections underwent a morphometric study to identify differences among experimental groups. Specifically, mean total pores number, pores average size and the % area occupied by the pores were considered (Figure 1B). Regarding the scaffolds surface, the pores number showed the following descending order ± standard deviation (SD): OxPVA + 10%P (871.60 ± 156.00) > OxPVA + 15%P (458.20 ± 73.50) > OxPVA + 25%P 398.20 ± 171.40 > OxPVA (88.60 ± 106.10). Among the porous scaffolds, significant differences were detected when comparing OxPVA + 10%P to OxPVA + 15%P and +25%P (*P* < 0.01). The average pore size increased with the gelatin concentration, with OxPVA + 25%P showing the largest pores (79.89 ± 26.98µm^2^) followed by OxPVA + 15%P (59.90 ± 6.62 µm^2^), OxPVA + 10%P (44.33 ± 11.56 µm^2^) and OxPVA (4.12 ± 1.39 µm^2^). Comparing porous scaffolds, a significant difference was found between OxPVA + 25%P and OxPVA + 10%P scaffolds (*P* < 0.01). The percentage of the area occupied by the pores was highest in OxPVA + 10%P (27.97 ± 5.57%) followed by OxPVA + 25%P (21.65 ± 5.16), OxPVA + 15%P (20.26 ± 4.05%) and OxPVA (0.30 ± 0.36%). All scaffolds incorporated with gelatin showed a significantly higher area occupied by pores compared to the control (*P* < 0.0001; Figure 1Ba–c). Focusing on the transversal sections, the pores number followed this descending order: OxPVA + 10%P (578.20 ± 182.30) > OxPVA + 15%P (475.20 ± 130.00) > OxPVA + 25%P (281.40 ± 37.60) > OxPVA (2.00 ± 2.55); among the porous scaffolds, the number of pores in OxPVA + 10%P was significantly higher than in OxPVA + 25%P (*P* < 0.01). The average size of the pores was largest in OxPVA + 25%P (109.30 ± 22.11µm^2^), followed by OxPVA + 15%P (41.70 ± 21.54µm^2^), OxPVA + 10%P (27.00 ± 5.61 µm^2^) and OxPVA (2.03 ± 1.93µm^2^). Pores in OxPVA + 25%P were significantly larger compared to those in OxPVA + 10%P and OxPVA + 15%P scaffolds (*P* < 0.0001). The percentage of the area occupied by pores was highest in OxPVA + 25%P (22.46 ± 2.61), followed by OxPVA + 15%P (13.87 ± 5.29%), OxPVA + 10%P (11.30 ± 3.35%) and OxPVA (0.004 ± 0.007%). Considering porous scaffolds, the percentage of the area occupied by pores in OxPVA + 25%P samples was significantly higher than that in both OxPVA + 10%P (*P* < 0.001) and OxPVA + 15%P scaffolds (*P* < 0.01; Figure 1Bd–f).

### Mechanical properties of porous scaffolds

**Figure 2.**
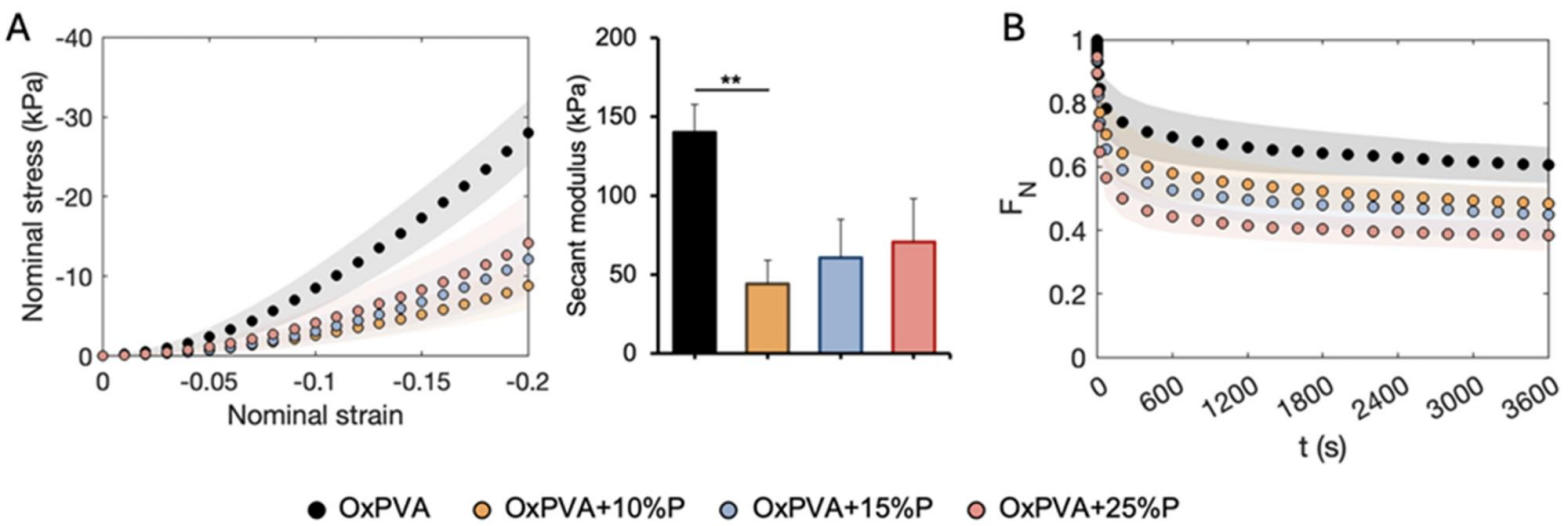
Compressive mechanical properties of OxPVA hydrogels with varying porosity percentage (0%, 10%, 15% and 25%). (**A**) Almost-instantaneous response: mean nominal stress ± standard deviation as a function of nominal strain, and comparison of compressive stiffness calculated as the secant modulus at 20% strain (**P < 0.01). (**B**) Viscoelastic response: normalized force over time.

Mechanical compression tests were performed to characterize the behavior of porous OxPVA hydrogels (i.e. OxPVA, OxPVA + 10%P, OxPVA + 15%P and OxPVA + 25%P) subjected to compressive strain, and investigate the influence of porosity over the mechanical strength. Figure 2A shows that the almost-instantaneous stress–strain response varied with porosity, with the lower porosity percentages exhibiting higher stresses at maximum compression. To assess significant differences among the porous hydrogels, the stiffness at 20% strain was calculated, as secant modulus (Figure 2A). Statistically significant reductions in stiffness, compared to the nonporous material, were observed only for OxPVA + 10%P samples (*P* = 0.004). Regarding force reduction over time due to consolidation process, the higher porosity was associated with the larger force decay (Figure 2B). Both at short (10 s) and long (3600 s) time points, the porous samples exhibited a greater reduction in force; however, this difference with respect to the nonporous scaffold was found to be statistically significant only in the case of OxPVA + 25%P (*P* = 0.024 and *P* = 0.006, respectively).

### Porous scaffolds cytocompatibility

**Figure 3.**
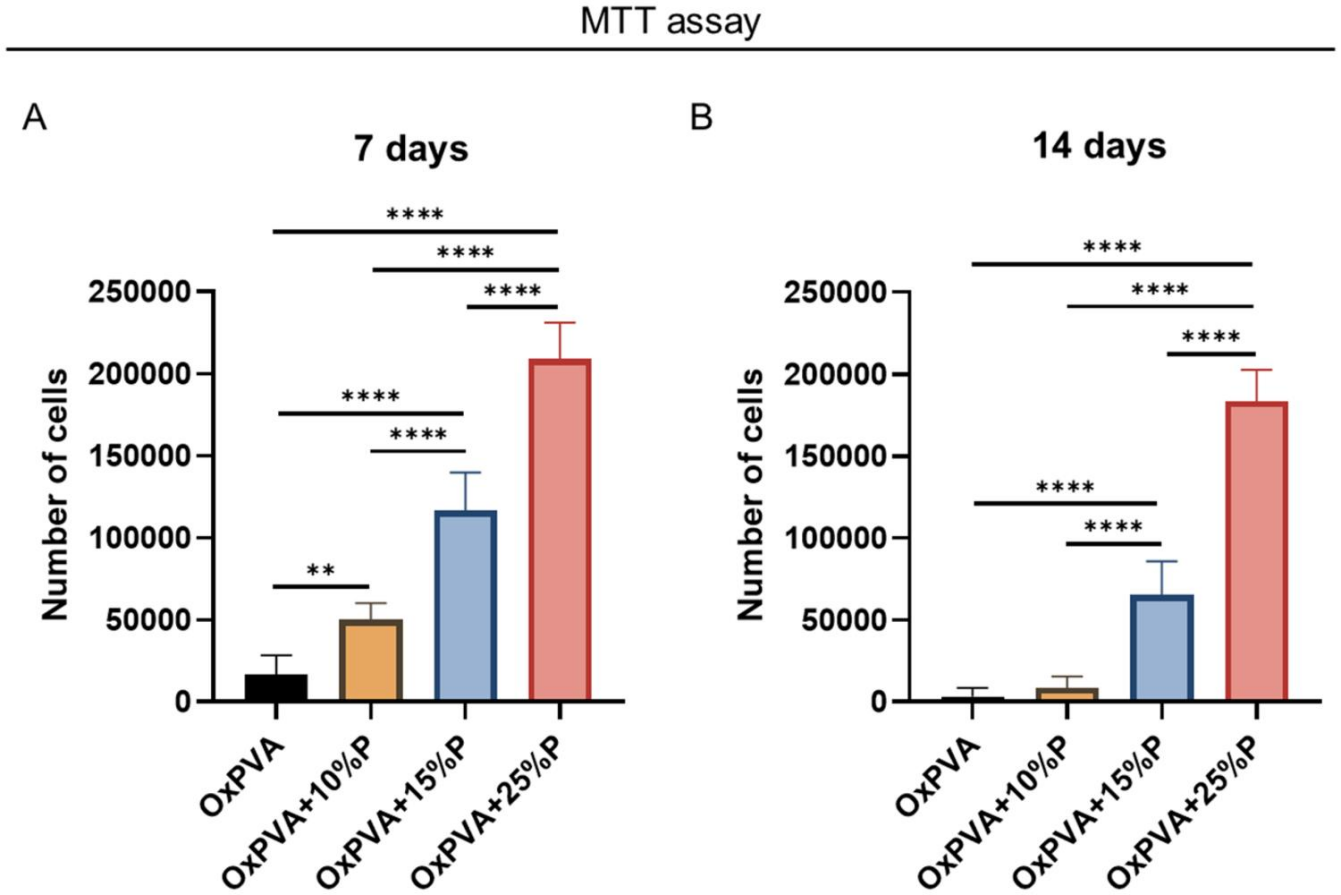
Human bone marrow mesenchymal stem cells (HM1-SV40) adhesion and proliferation on different OxPVA-based scaffolds prepared without porogen (OxPVA) or with different porogen (P) percentages (OxPVA + 10%P, OxPVA + 15%P, OxPVA + 25%P). Cells behavior was determined by MTT assay at 7 days (**A**) and 14 days (**B**) from seeding. (**P *<* 0.01; ****P *<* 0.0001).

Eventual differences in HM1-SV40 cells proliferation were analyzed at 7 and 14 days from seeding the scaffolds. At Day 7, cells adhesion and proliferation on smooth OxPVA (control) was almost negligible (*P* *<* 0.01 vs OxPVA + 10%P; *P* *<* 0.0001 vs OxPVA + 15%P and +25%P); whereas, comparing the porous scaffolds, it was identified a significantly higher cells proliferation rate on OxPVA + 25%P supports compared to the other two porous groups (*P* < 0.0001). Additionally, significantly more cells grew over OxPVA + 15%P versus OxPVA + 10%P (*P* < 0.0001; Figure 3A).

After 14 days of culture, cells proliferation showed a decrease in the whole cohort and, while their number was significantly higher in OxPVA + 25%P and OxPVA + 15%P samples versus control group (*P* < 0.0001), the difference was no longer detectable whether comparing OxPVA to OxPVA + 10%P supports. Furtherly, OxPVA + 25%P scaffolds showed a significantly higher number of cells compared to both OxPVA + 15%P and OxPVA + 10%P samples (*P* < 0.0001); a significant difference was also highlighted comparing OxPVA + 15%P to OxPVA + 10%P (*P* < 0.0001; Figure 3B).

### Decellularization of articular cartilage

#### Gross appearance and ultrastructure

It is well established that cartilage tissue has a dense ECM that could hinder the decellularization solutions penetration; hence, the tissue was finely minced to facilitate the decellularization process by increasing the contact area with the solutions.

**Figure 4.**
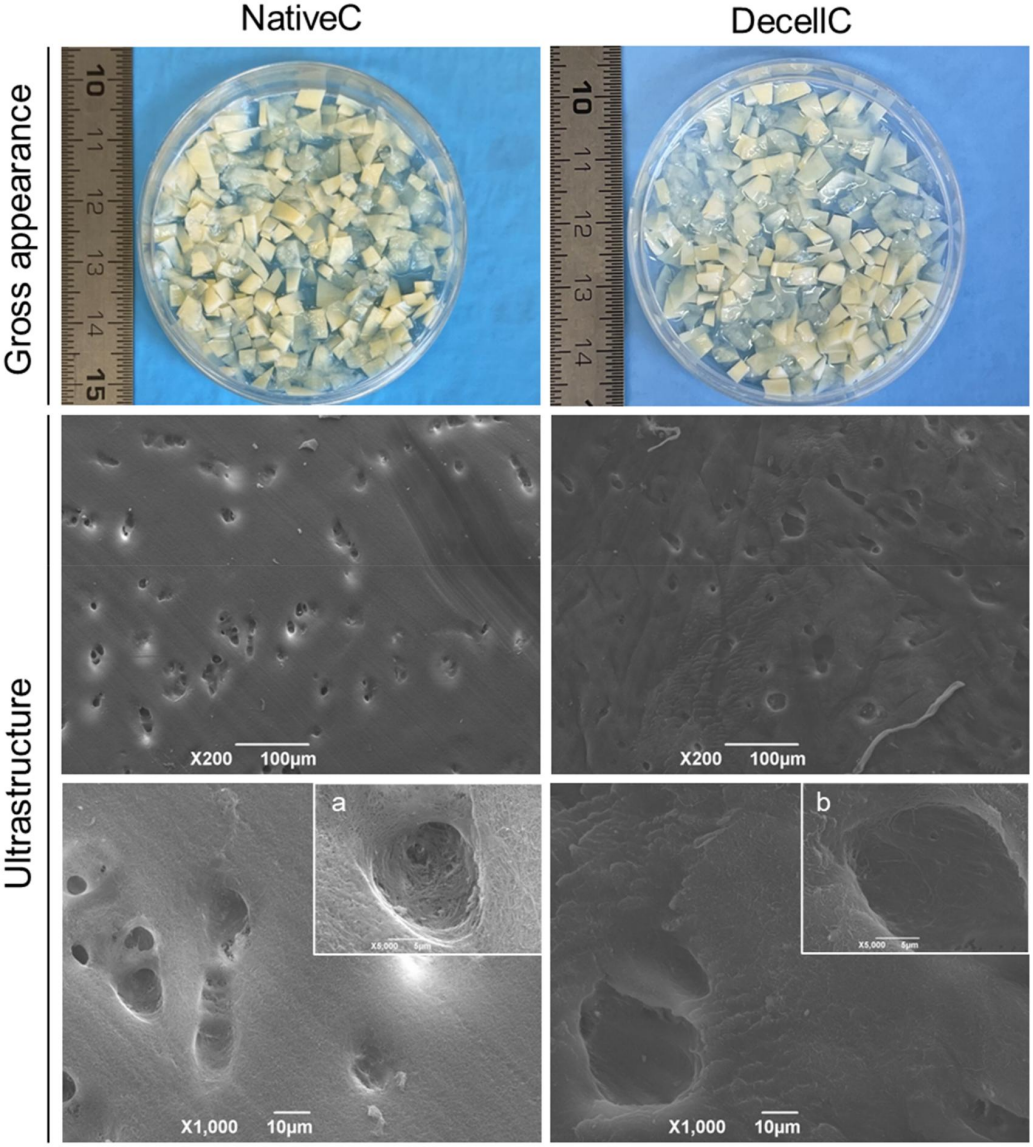
Articular cartilage gross appearance and ultrastructure by SEM before (native cartilage, NativeC) and after decellularization (decellularized cartilage, DecellC; scale bars: 100 µm; 10 µm). Lacune magnification is showed within the inserts (a, b; scale bar: 5 µm).

Observing samples gross appearance, the specimens appeared as yellowish in the native tissue acquiring a lighter color (whiteish) after decellularization (Figure 4).

SEM analysis of native and decellularized cartilage was performed to compare the ultrastructural characteristics of the samples’ surface. Native tissue was more compact than the decellularized one; this was particularly evident focusing on the lacunae profiles that were clearly identifiable in both the groups but appeared as swelled after decellularization (Figure 4).

#### Nuclei removal verification and residual DNA quantification

**Figure 5.**
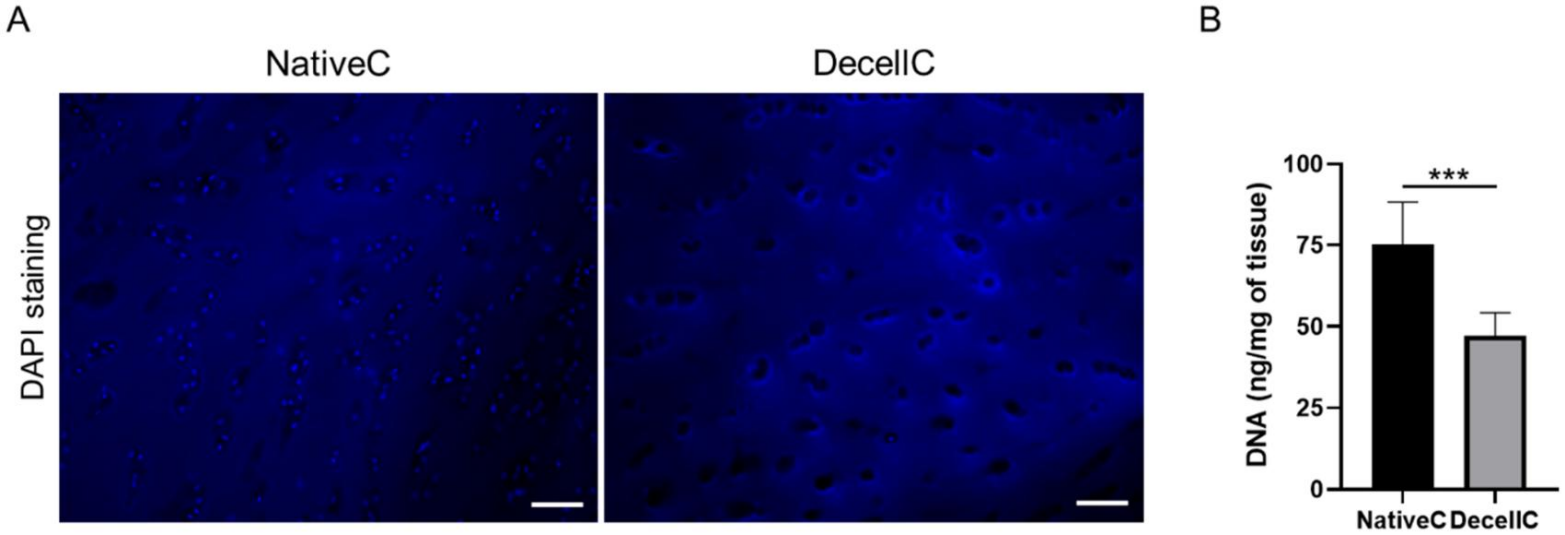
Evaluation of decellularization protocol effectiveness. (**A**) DAPI staining of native and decellularized cartilage (NativeC and DecellC, respectively); nuclei appear as blue, fluorescent dots (scale bar: 50 µm). (**B**) Quantification of total DNA in NativeC and DecellC (***P *<* 0.001).

Articular cartilage was successfully decellularized through three detergent-enzymatic cycles, as corroborated by DAPI fluorescent nuclear staining and DNA quantification. Regarding DAPI staining (Figure 5A), native cartilage distinguished for the presence of several cells’ nuclei (blue dots) not detectable following decellularization; in fact, decellularized tissue only showed dark lacunae.

DAPI staining results were supported by DNA quantification: as shown in Figure 5B, decellularized cartilage displayed a reduction of 37.4% (47.13 ± 7.14 ng/mg) in DNA content versus native cartilage (75.28 ± 13.01 ng/mg; *P* < 0.001).

Once it was confirmed decellularization process success, decellularized ECM was characterized for specific proteins distribution/content and then homogenized to allow composite scaffolds fabrication.

#### ECM characterization

Collagen (total collagen and collagen type I/III) and GAGs were focused through histological stainings to evaluate decellularization impact. Specifically, Azan–Mallory staining (blue color) suggested the presence of similar total collagen distribution in both native and decellularized tissue. Additionally, red-stained nuclei were present in native cartilage lacunae only, furtherly confirming DAPI staining results.

Picrosirius Red staining highlights the natural birefringence of collagen fibers under polarized light; collagen type I fibers appear in yellow to orange, whereas collagen type III fibers appear in green. Collagen type I was similarly detected in both native and dECM; collagen type III was nearly not present.

**Figure 6.**
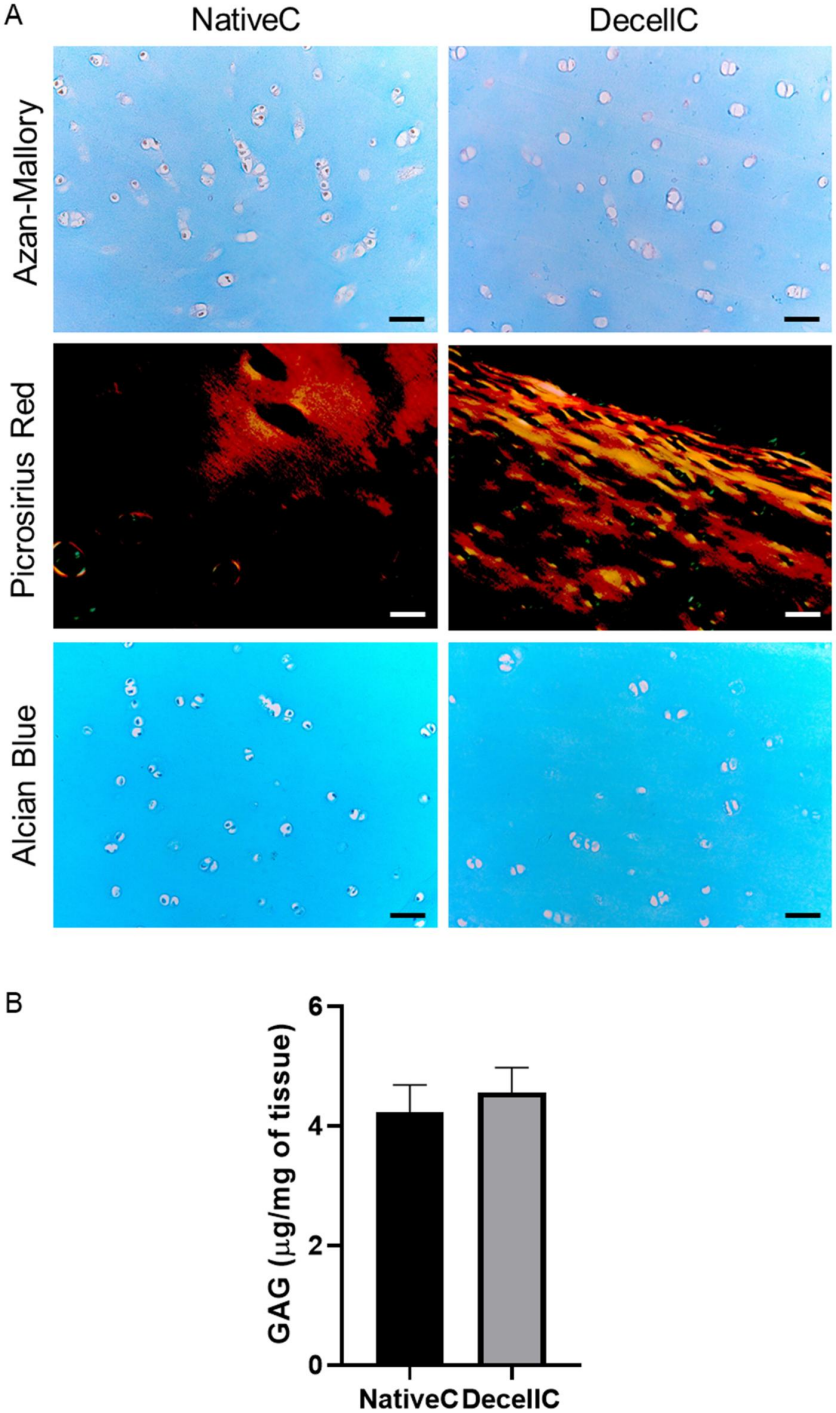
Evaluation of ECM proteins. Specifically, native cartilage (NativeC) and decellularized cartilage (DecellC) were compared for total collagen appearing as light blue (Azan–Mallory staining); collagen type I (red–yellow)/III (green; picrosirius red staining and observation under polarized light); glycosaminoglycans (GAGs) appearing as light blue (Alcian Blue staining; scale bar: 50 µm). (B) Quantification of GAGs content in NativeC and DecellC samples. (***P < 0.001).

Alcian blue staining, highlighting GAGs in blue, showed a similar color distribution in both native and decellularized tissues. Again, blue-stained nuclei were recognized within lacunae of native cartilage (Figure 6A).

To furtherly support Alcian Blue staining results, GAGs quantification was performed through a biochemical assay. As reported in Figure 6B, decellularized cartilage showed a slightly higher GAGs content (4.56 ± 0.41 µg/mg) compared to native cartilage (4.23 ± 0.46 µg/mg), however, this difference was not statistically significant.

#### Composite porous scaffolds characterization

**Figure 7.**
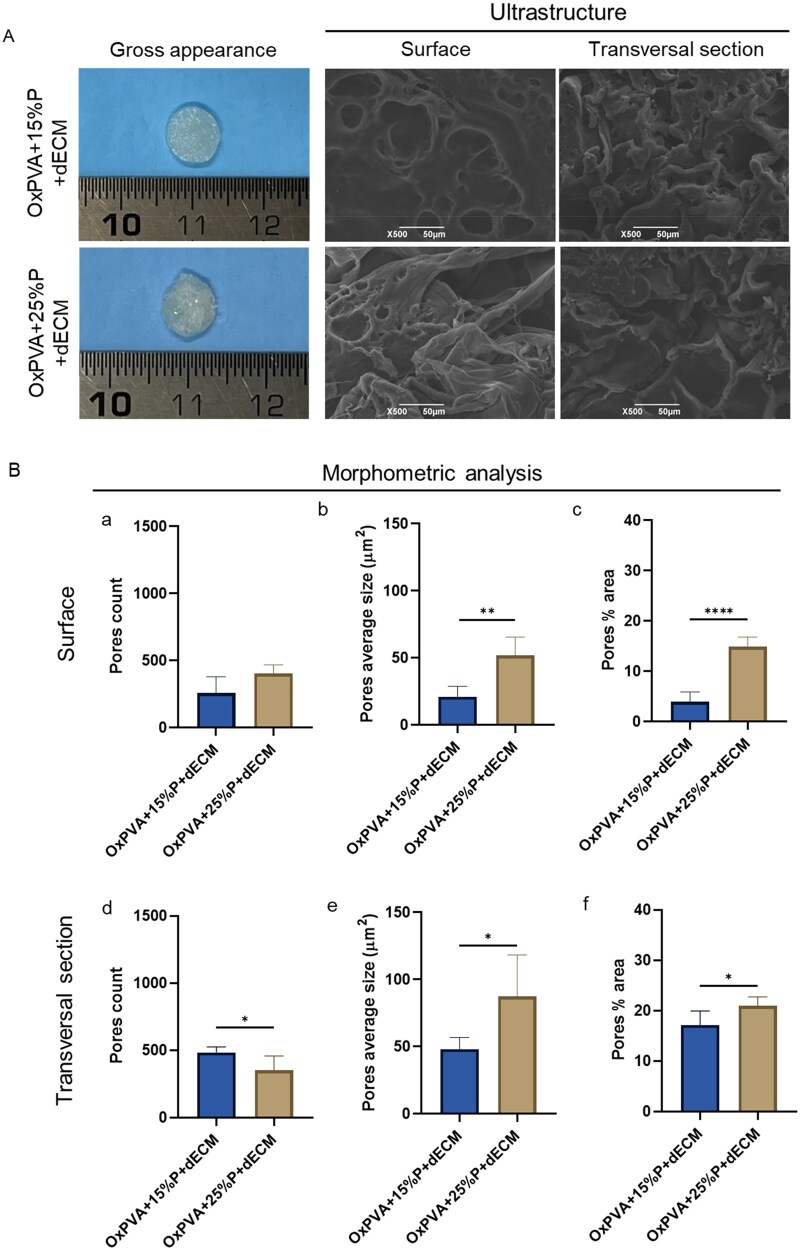
Porous scaffolds combined with acellular matrix comparison. (**A**) Gross appearance and ultrastructure by SEM of OxPVA-based scaffolds+decellularized extracellular matrix (dECM). OxPVA porous scaffolds fabricated with different percentages of porogen (P): OxPVA + 15%P + dECM and OxPVA + 25%P + dECM. Scaffolds surface and transversal section were both considered (scale bars: 50 µm). (**B**) Porosity assessment and morphometric study. Analyzing SEM photomicrographs, surface and transversal sections of the scaffolds were compared for pores count (B, a, d), pores average size (B, b, e) and pores % area (B, c, f). (*P *<* 0.05; **P *<* 0.01; ****P *<* 0.0001).

To furtherly increase the bioactivity of OxPVA porous scaffolds, a weighted amount of dECM suspension (25% w/w to OxPVA) was mechanically incorporated within the mixtures: OxPVA + 15%P and OxPVA + 25%P, respectively. Once crosslinked the hydrogel and removed the porogen, the gross appearance of scaffolds was preliminarily compared. OxPVA + 25%P+dECM scaffolds appeared as more swelled than OxPVA + 15%P+dECM which, in turned, showed to better maintain the discoidal shape given by the mold. SEM micrographs were obtained to characterize the superficial and transversal ultrastructure of the scaffolds; the comparison showed that the pores were larger in OxPVA + 25%P+dECM than in OxPVA + 15%P+ECM (Figure 7A). Thus, a morphometric study was conducted to better describe composite scaffolds organization and investigate the impact of homogenized dECM addition over scaffolds the porosity. Superficial and transversal porosity was analyzed in terms of number of pores, average size and percentage of area occupied by them.

Considering the scaffolds surface, the number of pores was comparable between the two groups with a mean pores number of 256.4 ± 123.2 and 401.4 ± 68.18 for OxPVA + 15%P+dECM and OxPVA + 25%P+dECM scaffolds, respectively. The measured average size of the pores was significantly higher in OxPVA + 25%P+dECM (51.90 ± 13.62 µm^2^) than OxPVA + 15%P+dECM (20.93 ± 7.9µm^2^; *P* < 0.01) and, similarly, also the area occupied by pores was higher in OxPVA + 25%P+dECM (14.90 ± 1.93%) than in OxPVA + 15%P+dECM (3.92 ± 1.96%; *P* < 0.0001).

Focusing on transversal sections, image-processing analysis revealed a significantly higher pores number in OxPVA + 15%P+dECM (483.00 ± 41.07) than in OxPVA + 25%P+dECM (350.6 ± 106.4; *P* *<* 0.05). As regards pores average size, pores were larger in OxPVA + 25%P+dECM (87.30 ± 31.01µm^2^) than in OxPVA + 15%P+dECM (48.16 ± 8.66µm^2^; *P* *<* 0.05) and, similarly, it was also the area occupied by pores: 17.12 ± 2.80% for OxPVA + 15%P+dECM and 20.90 ± 1.81% for OxPVA + 25%P+dECM scaffolds (*P* < 0.05; Figure 7B).

**Figure 8.**
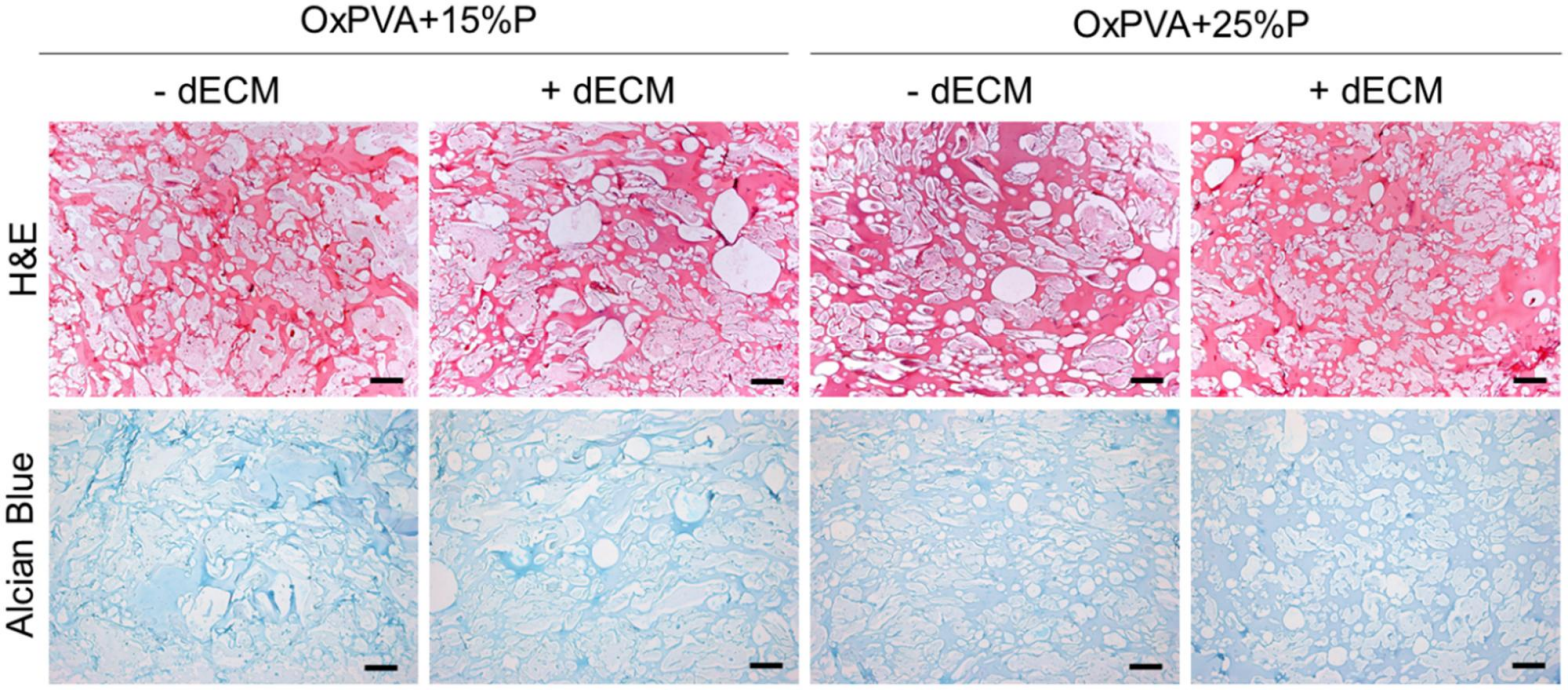
Histological analysis of porous OxPVA scaffolds prepared with different percentages of porogen (P; OxPVA + 15%P; OxPVA + 25%P) without/with decellularized extracellular matrix (±dECM) stained with hematoxylin & eosin (H&E) and Alcian Blue (scale bar: 200 µm).

The incorporation of dECM within the porous scaffolds is expected to enhance their bioactivity. To characterize the distribution of ECM-derived macromolecules within the porous OxPVA + dECM supports, histological staining was performed. Considering the use of ECM-derived protein suspension, the histological analysis would eventually reveal a modification in color intensities instead of specific tissue-related structures (Figure 8). Preliminarily, organization of porous OxPVA scaffolds ± dECM was evaluated by H&E. No evident structural differences were highlighted; occasionally, gelatin residues were identified between empty spaces. Alcian blue staining did not show any differences in terms of color shade or intensity, a diffuse blue colour was recognizable.

#### Mechanical properties of porous scaffolds with dECM

**Figure 9.**
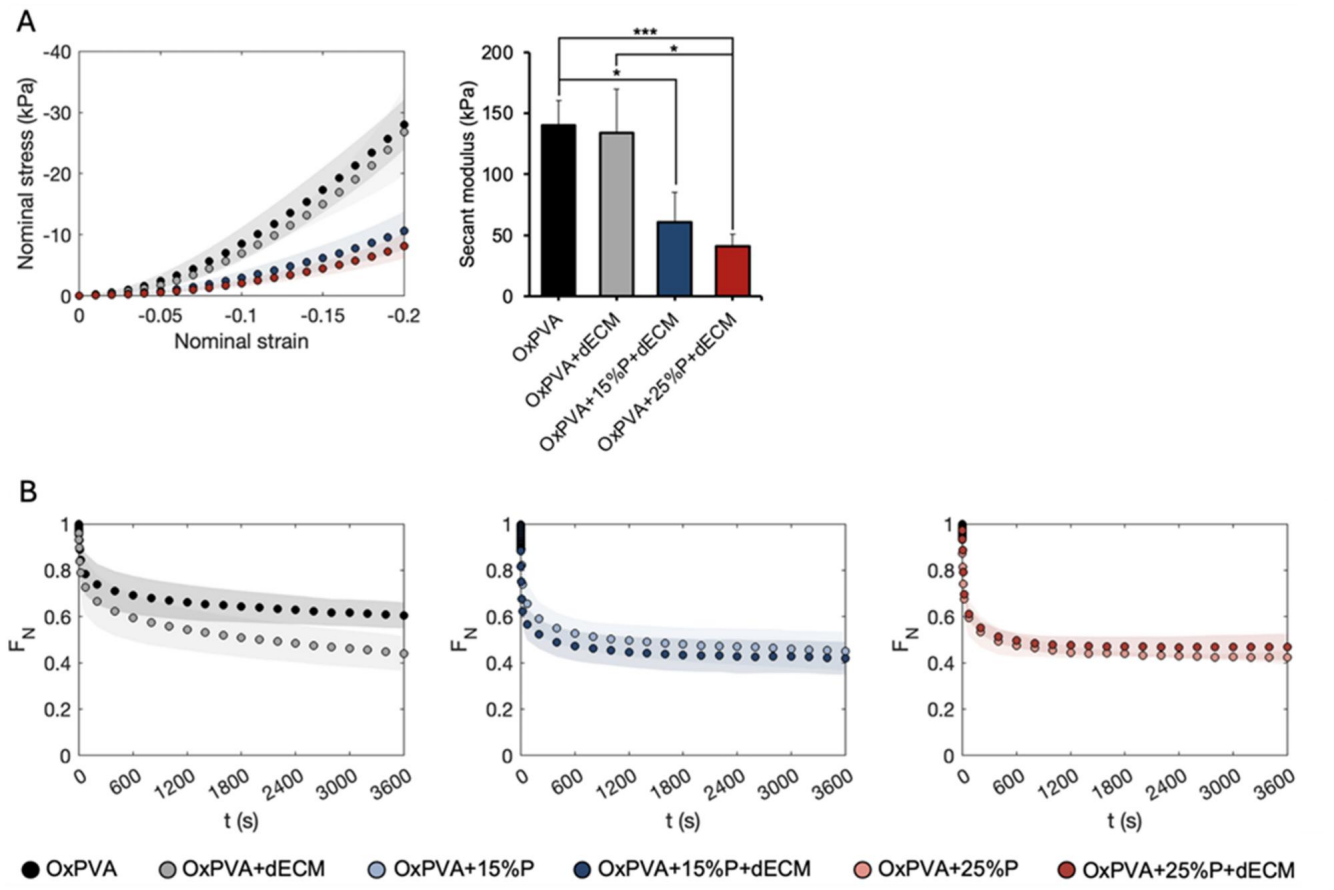
Compressive mechanical properties of composite OxPVA hydrogels containing dECM, with different percentage of porosity (0%, 15%, 25%). (**A**) Almost-instantaneous response: mean nominal stress ± standard deviation as a function of nominal strain, and comparison of compressive stiffness calculated as the secant modulus at 20% strain (* *P* < 0.05, *** *P* < 0.005). (**B**) Viscoelastic response: normalized force reduction over time, comparing porous hydrogels with and without dECM incorporation.

The effect of incorporating dECM into porous scaffolds on their mechanical properties was evaluated through almost-instantaneous compression and consolidation tests. The behavior of the stress–strain curves (Figure 9A) remained unaltered following dECM incorporation, and no reduction in stiffness could be attributed to the presence of the matrix. Consistently with the behavior observed in porous scaffolds without dECM, stiffness decreased significantly with increasing porosity (Figure 9A). In contrast, dECM incorporation altered the viscoelastic response of the continuous material both at short (10 s) and long (3600 s) term, with a significant force reduction (*P* = 0.030 for both; Figure 9B). However, introducing porosity eliminates the difference observed in the nonporous material.

#### FRAP analyses

**Figure 10.**
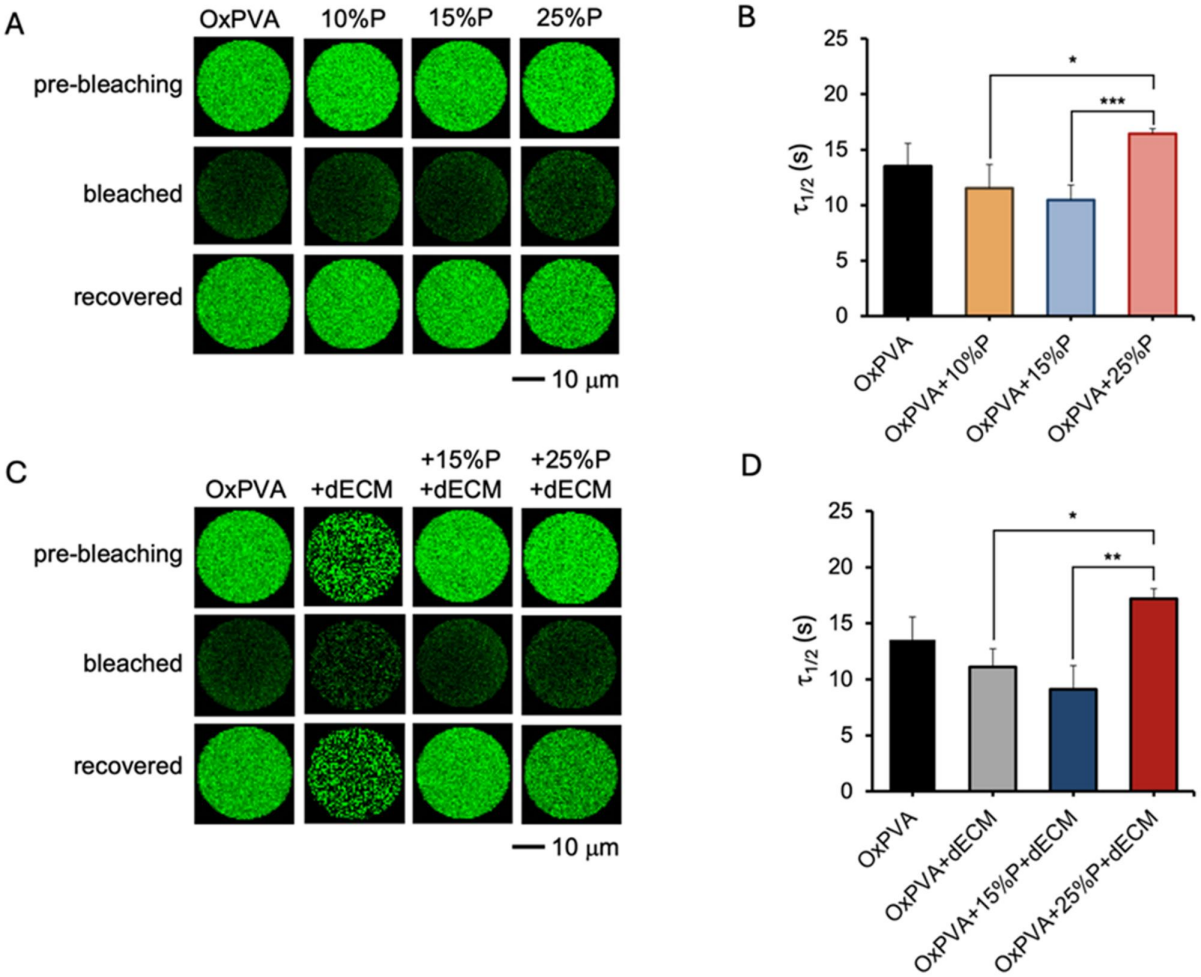
FRAP analysis. (**A**) Representative pre-bleaching, post-bleaching and 30 s postbleaching images of the circular ROI for all porous hydrogel conditions and the nonporous OxPVA control. (**B**) Half recovery time (τ_1/2_) for all porous hydrogels. (**C**) Representative pre-bleaching, postbleaching and 30 s postbleaching images of the circular ROI for composite OxPVA hydrogels containing dECM with porosity percentage of 0%, 15% and 25%, along with the nonporous OxPVA control. (**D**) Half recovery time (τ_1/2_) for composite OxPVA hydrogels + dECM. *P < 0.05, **P < 0.01 scale bars: 10 µm.

OxPVA hydrogels diffusion properties were assessed by FRAP using 500 kDa fluorescence-labeled dextran. Photobleaching reduced fluorescence intensity within the ROI (Figure 10A and C), followed by progressive recovery due to molecular diffusion from surrounding regions. The half-recovery time (τ_1_/_2_) decreased with 10% and 15% porosity but increased, though not significantly, at 25% (Figure 10B). Porous composite scaffolds exhibited the same trend (Figure 10D). Incorporation of dECM slightly reduced τ_1_/_2_, without statistical significance.

#### Porous composite scaffolds cytocompatibility

To assess composite scaffolds’ cytocompatibility, supports were seeded with 100 000 HM1-SV40 and cell proliferation was evaluated after 7 and 14 days.

**Figure 11.**
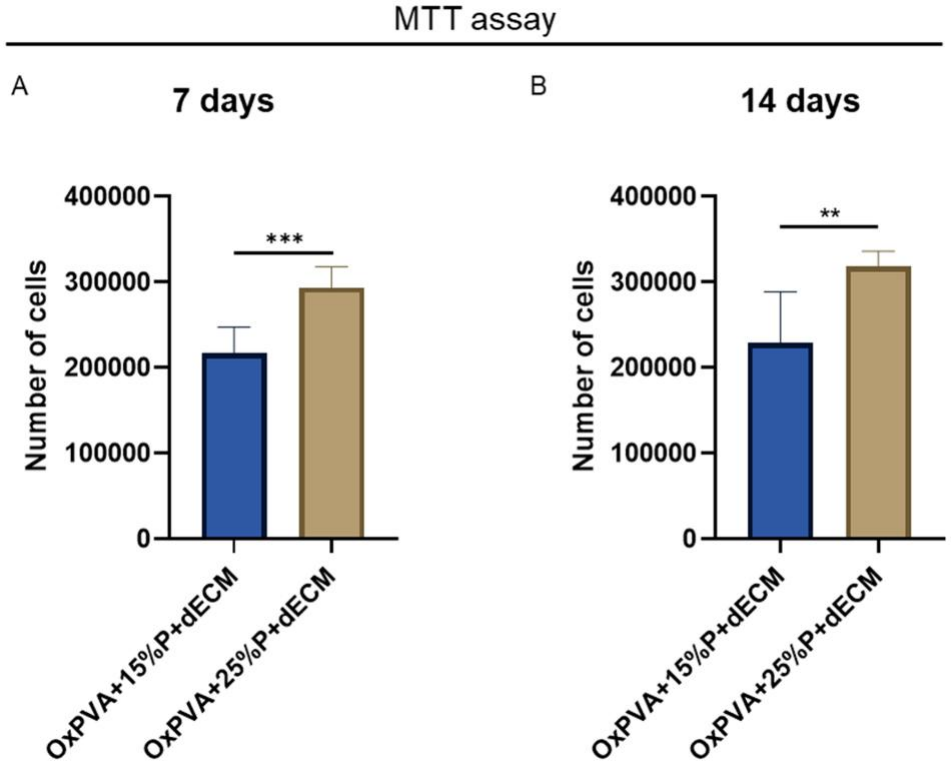
Human bone marrow mesenchymal stem cells (HM1-SV40) adhesion and proliferation on different OxPVA-based scaffolds prepared with different porogen (P) percentages (OxPVA + 15%P, OxPVA + 25%P) in presence of decellularized extracellular matrix (dECM). Cells behavior was determined by MTT assay at 7 days (**A**) and 14 days (**B**) from seeding (**P *<* 0.01; ***P *<* 0.001).

After seven days of culture, cells showed a significantly higher proliferation on OxPVA + 25%P + dECM than OxPVA + 15%P + dECM (*P* *<* 0.001). At Day 14, cell proliferation remained stable, and a higher cells number was still detectable on OxPVA + 25%P + dECM (*P* *<* 0.01; Figure 11A and B).

#### Bioactive supports induce time sequence gene expression

To assess the biological activity of the bioactive supports, RNA was extracted from cells cultured for 7 or 14 days. Expression of genes related to collagen formation, cartilage differentiation and remodelling was determined by quantitative PCR. Results are reported in Figure 12. The expression of COL2A1 mRNA transcripts increased by 1.34-fold in OxPVA 25%P and by 1.26-fold in OxPVA 25%P+ECM. A 1.5-fold increase was also observed in OxPVA 15%P+ECM. Smaller increases in COL9A1 and COL10A1 gene expression were detected in all tested bioactive supports. After 14 days of culture, COMP gene expression increased by 1.52-fold in OxPVA 15%P+ECM, 1.35-fold in OxPVA 25%P and by 1.26-fold in OxPVA 25%P+ECM. SOX9 transcript levels increased in cells cultured for 14 days in OxPVA 25%P+ECM, while SOX5 expression increased over time without differences among the supports. The expression of prolyl-4-hydroxylase alpha 2 (P4HA1 gene) progressively increased over the culture period and reached its highest level in cells grown on ECM-enriched supports at 14 days. Regarding genes involved in matrix remodelling, all bioactive matrices supported the expression of MMP16 and MMP14, with no clear time-dependent variations (Supplementary Table 1).

**Figure 12.**
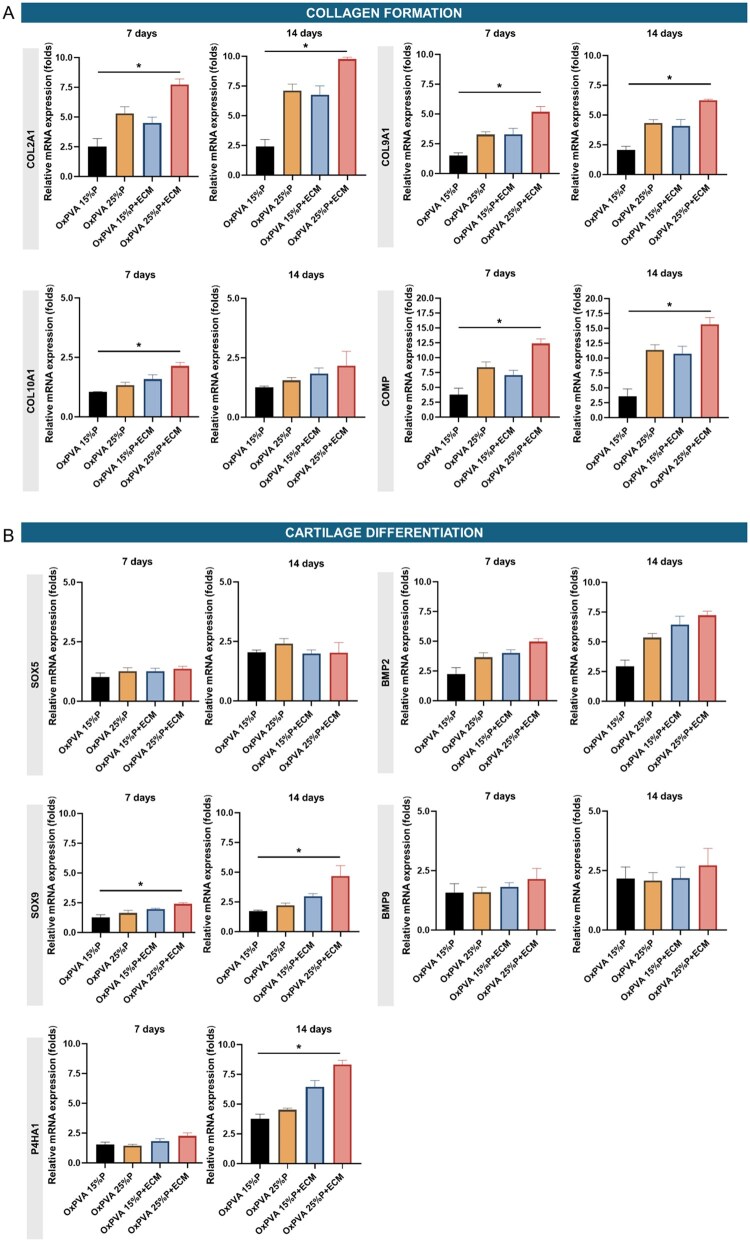
qRT-PCR analysis of multiple genes expression from HM1-SV40 cells seeded and cultured for 7 and 14 days on the four different scaffolds. Genes involved in collagen formation (**A**), cartilage differentiation and remodelling (**C**) were considered (*P *<* 0.05).

**Figure 12.**
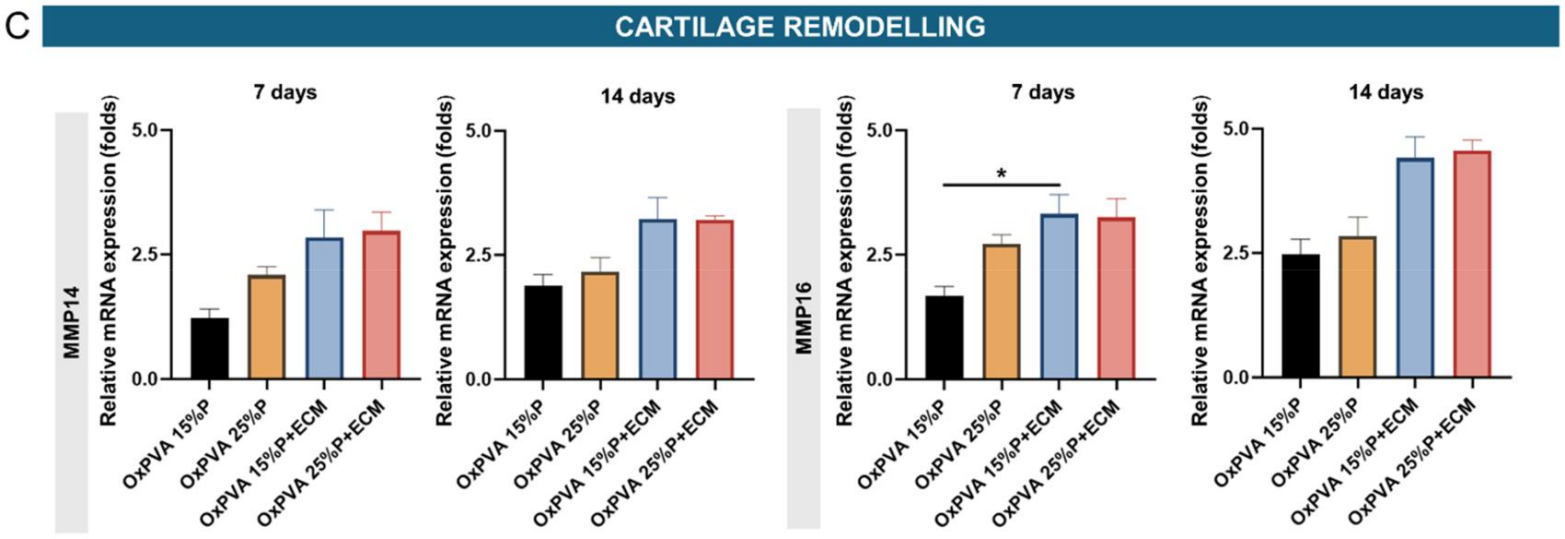
(Continued)

#### Semi-quantification of secreted proteins

**Figure 13.**
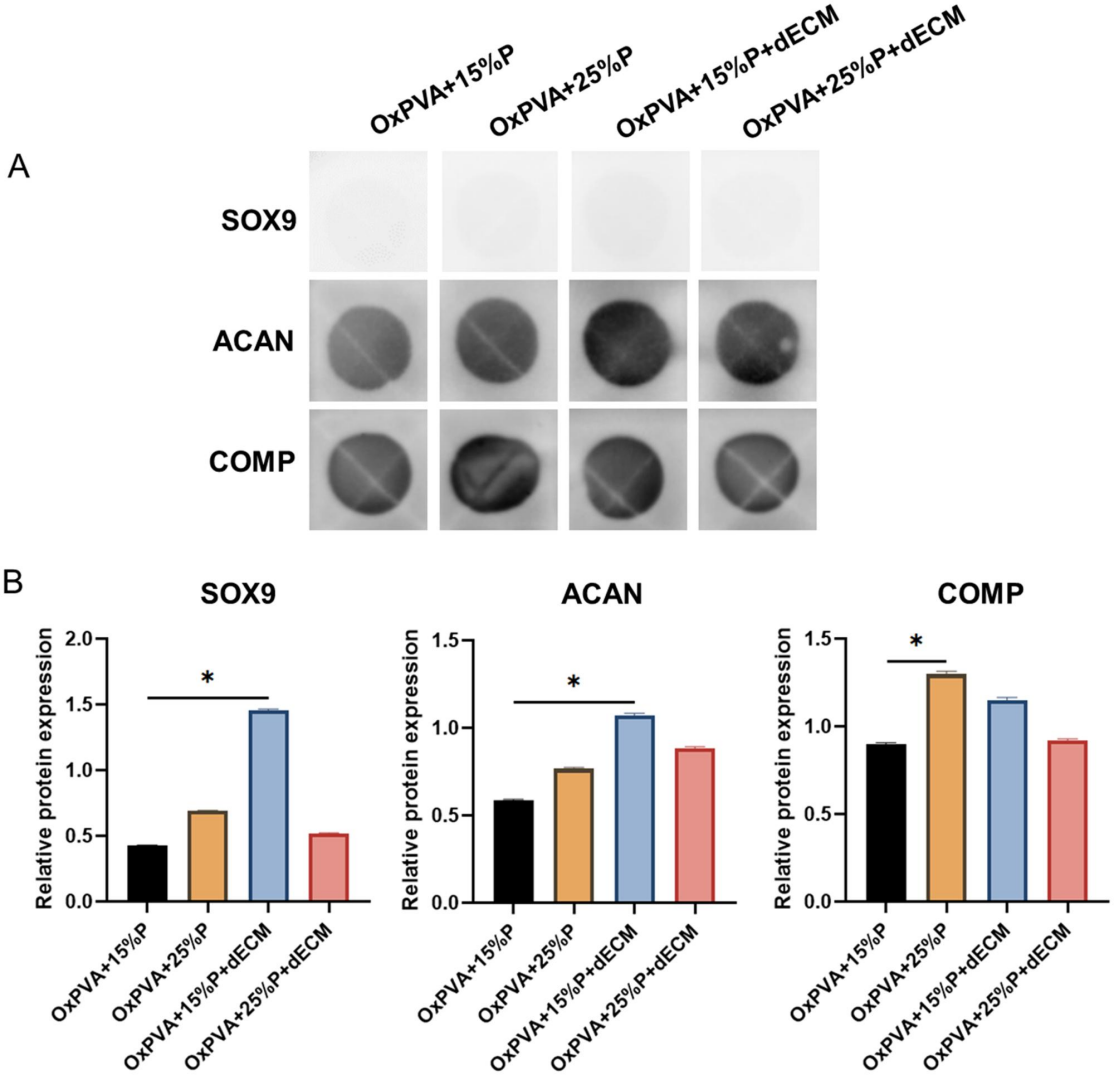
Dot blot analysis of cartilage-specific protein expression. (**A**) Representative dot blot images showing SOX9, ACAN and COMP expression in cells cultured for 14 days on the different scaffold formulations (OxPVA + 15%P, OxPVA + 25%P, OxPVA + 15%P + dECM and OxPVA + 25%P + dECM). (**B**) Densitometric quantification of relative protein expression levels normalized to subconfluent cells as internal control (*P < 0.05).

Dot blot assays allowed to evaluate the chondrogenic induction promoted by the different scaffold formulations through the analysis of cartilage-specific protein expression. After 14 days of cell culture on the scaffolds, immunodetection revealed the presence of SOX9, ACAN and COMP as distinct circular spots on the membranes for all the tested groups (Figure 13A). The densitometric analysis of the dot blot signals was performed to obtain the relative protein expression levels for SOX9, ACAN and COMP, normalized to the internal control (subconfluent cells; Figure 13B).

Regarding SOX9 and ACAN, the incorporation of dECM into the OxPVA + 15%P scaffolds significantly promoted the expression of these cartilage-specific markers (*P* < 0.05). Interestingly, a trend toward increased ACAN expression following the incorporation of cartilage matrix was observed also for OxPVA + 25%P supports, although the difference was not statistically significant. In parallel, COMP expression appeared to be significantly upregulated by the increase in porosity (*P* < 0.05), while the effect of dECM was detectable only in the OxPVA + 15%P scaffolds, although without reaching statistical significance.

#### Biocompatibility assessment in Sprague–Dawely rats

Ideal scaffolds should be biocompatible to minimize immune host reactions and inflammation, once implanted *in vivo*. To verify that, porous OxPVA ± dECM scaffolds were implanted in a dorsal subcutaneous pouch in Sprague–Dawley rats.

None of the implanted rats were euthanized or died prematurely before the experimental endpoint. Daily surveillance of the rats in the weeks after surgery did not show systemic signs of infection, neither local sign of rejection nor inflammation. After 14 days of *in vivo* implantation, rats were euthanized, and preliminary evidence was collected. The implanted scaffolds were surrounded by a thin fibrotic capsule, and upon dissection, no evident changes in size or integrity were observed compared to the pre-implantation samples’ gross appearance.

**Figure 14.**
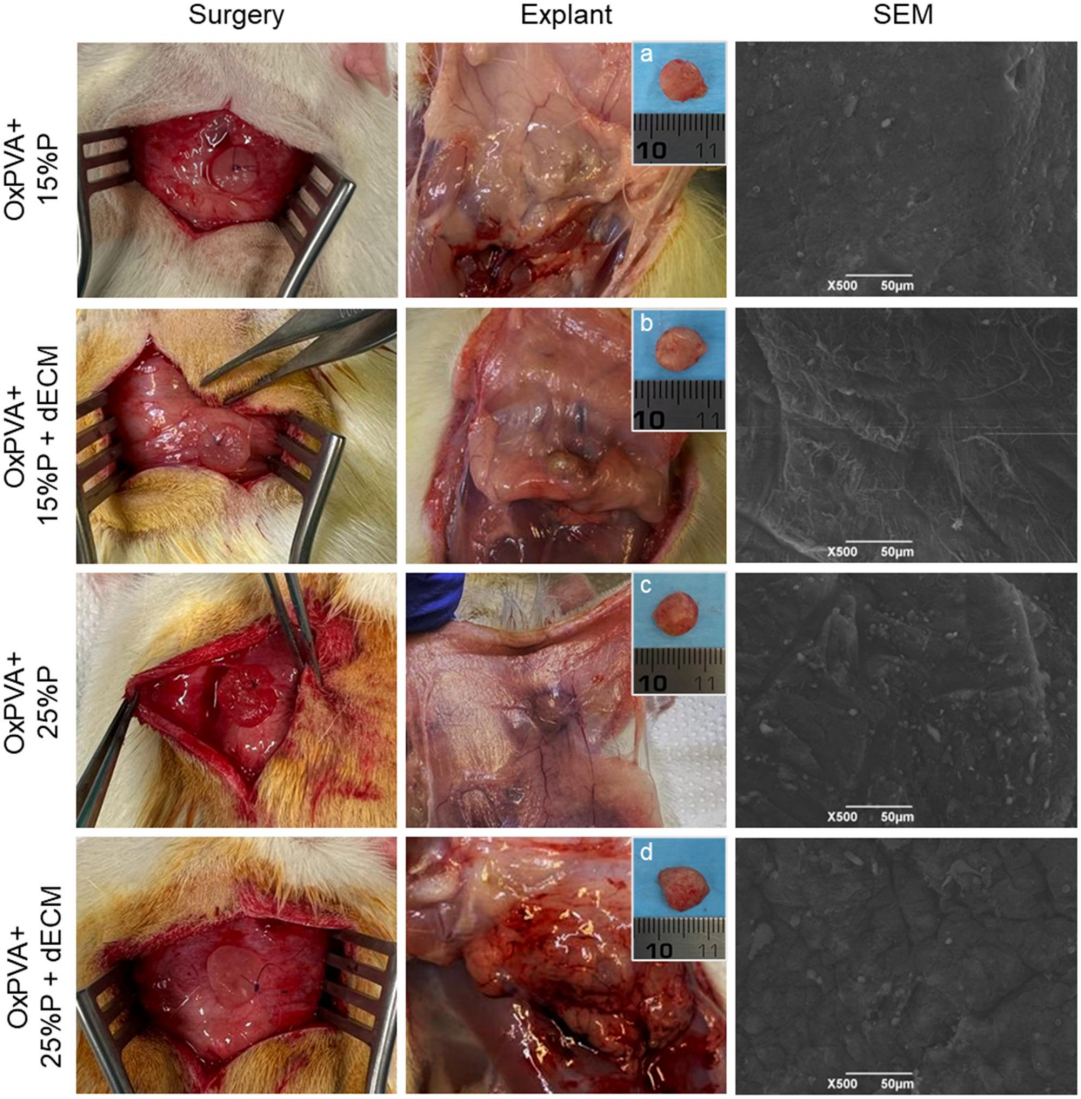
*In vivo* biocompatibility of porous OxPVA scaffolds without/with decellularized extracellular matrix (dECM). Dorsal subcutaneous implantation of OxPVA-based scaffolds in Sprague–Dawley rats; specimens’ explant after 14 days (**A**–**D** show explants after dissection) and subsequent ultrastructural analysis by scanning electron microscopy (SEM). Scale bar: 50 µm.

According to SEM images of explanted scaffolds, it was possible to recognize the fibrous nature of the capsule enclosing the samples, supporting the previous observations. Additionally, red and white blood cells, characterized by their biconcave disc and roundish appearance, were identified on the surface of the samples (Figure 14).

Histological analysis of the explants with the surrounding tissues was performed to further investigate the *in vivo* behavior of porous OxPVA ± dECM scaffolds.

H&E staining (Figure 15A) allowed to recognize the presence of the sample surrounded by the *panniculus carnosus* (upper side) and *latissimus dorsi* muscle (lower side, not shown). Porous scaffolds appeared as enveloped within a thin tissue-capsule penetrating the 3D structure of the supports.

**Figure 15.**
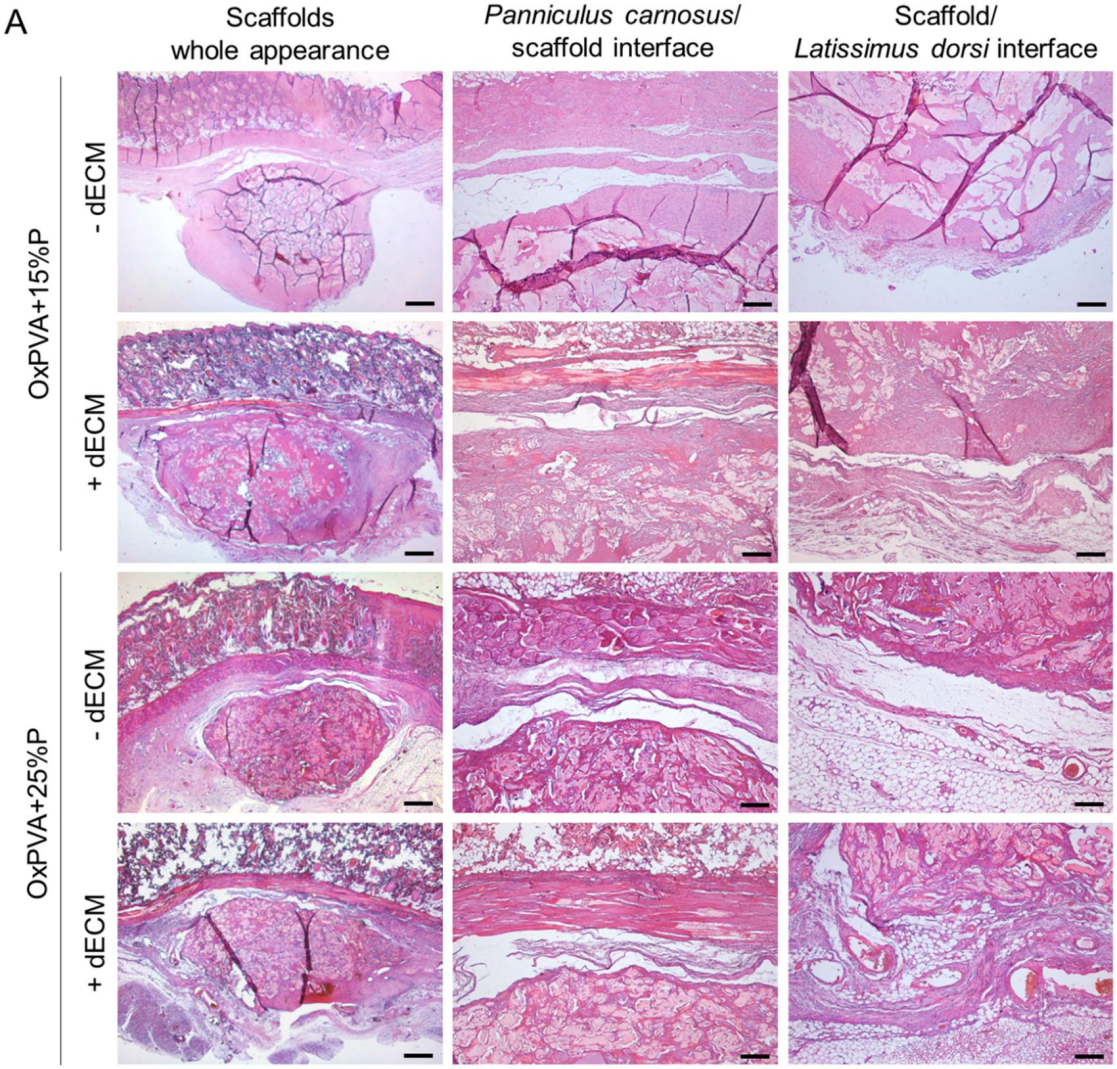
Histological characterization of OxPVA-based scaffolds following explant. OxPVA porous scaffolds prepared with different percentages of porogen (OxPVA + 15%P; OxPVA + 25%P), eventually combined with decellularized extracellular matrix (dECM) were stained with haematoxylin & eosin (**A**) and Azan–Mallory staining (collagen and fibro-connective tissue infiltration/distribution in blue) (**B**). Together with scaffolds overall appearance (scale bar: 800 µm), *panniculus carnosus*/scaffold interface and scaffold/latissimus dorsi interface were focused (scale bar: 200 µm).

**Figure 15.**
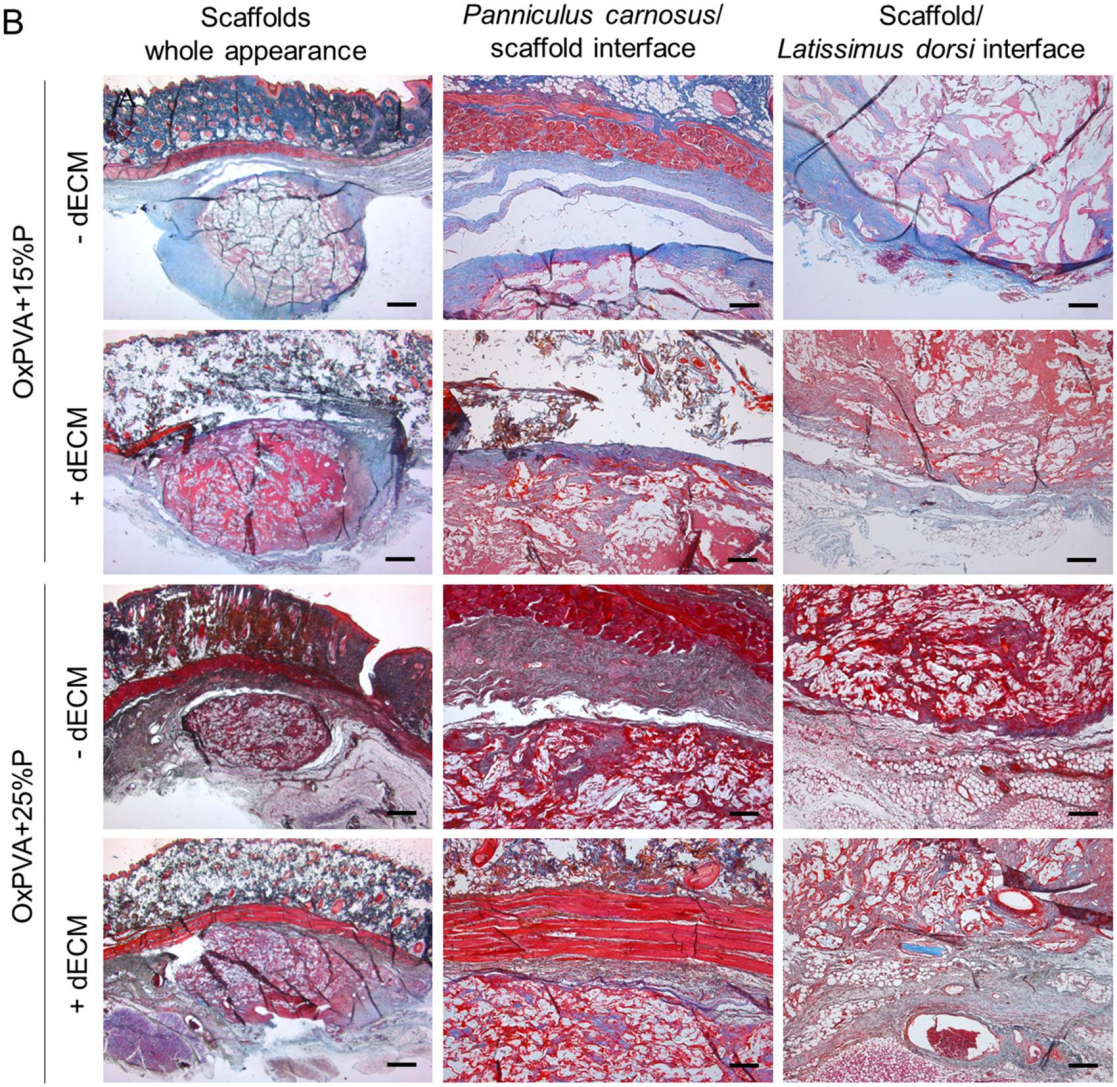
(Continued)

Azan–Mallory staining (Figure 15B) further characterized the organization of the capsule, suggesting the presence of fibro-connective tissue around the samples, stained in blue.

**Figure 16.**
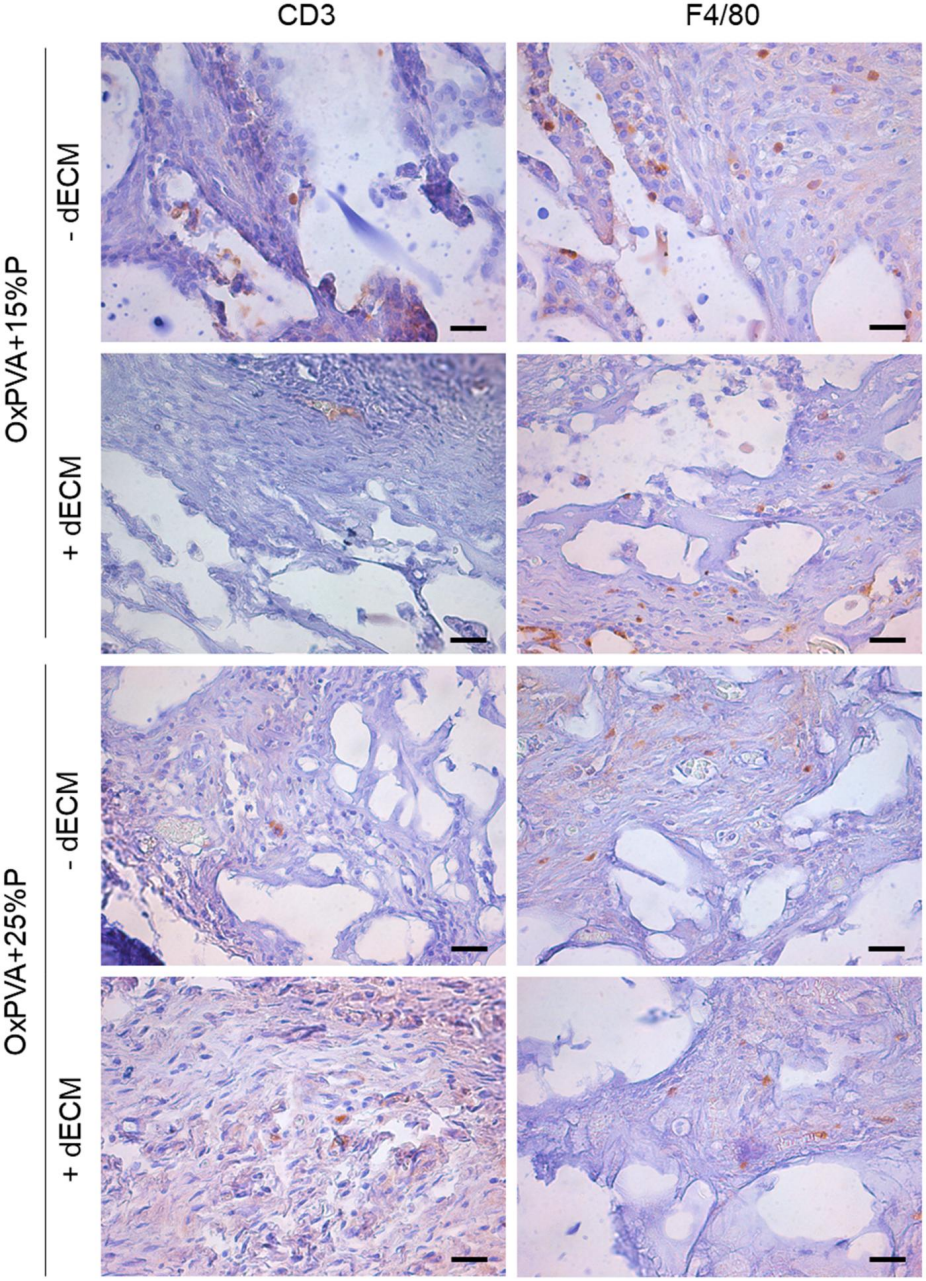
OxPVA-Based scaffolds explant immunohistochemical characterization. OxPVA porous scaffolds prepared with different percentages of porogen (OxPVA + 15%P; OxPVA + 25%P), eventually combined with decellularized extracellular matrix (dECM) were immunostained with CD3 for lymphocytes and F4/80 for macrophages detection. Scale bar: 25 µm.

Immunohistochemical staining with anti-CD3 and anti-F4/80 antibodies allowed to identify the presence of lymphocytes and macrophages in the explants, respectively. As shown in Figure 16, no severe inflammatory reactions were detected, although mild lymphocytic and macrophage infiltration, compatible with the type of surgery, was reported in all the samples (in brown).

### PLA scaffolds printing and bioactivity assessment *in vitro*

#### PLA scaffolds morphological and ultrastructural characterization

PLA was chosen for the fabrication of scaffolds intended to mimic the bone side of the osteochondral interface using the FDM technique. Open and interconnected pores are crucial for tissue regeneration, hence, trying to meet these requirements, three different geometries were designed.

**Figure 17.**
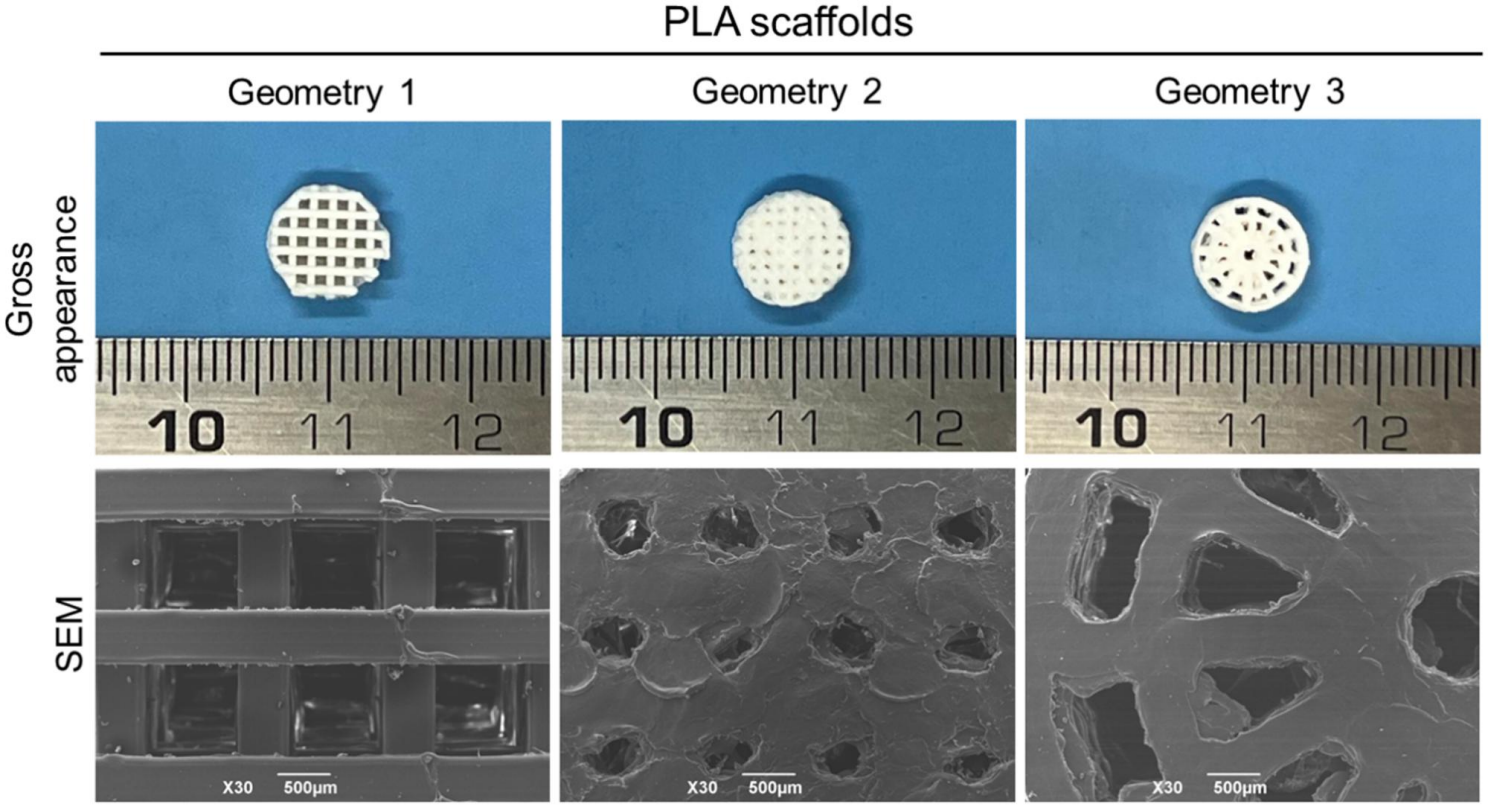
PLA scaffolds. Gross appearance of the 3D printed PLA scaffolds; investigation of the specific ultrastructure by scanning electron microscopy (SEM). scale bar: 500 µm.

Once 3D-printed, PLA scaffolds needed accurate removal of polymer printing residues. The resulting 3D-printed scaffolds are shown in Figure 17. Compared to Geometries 1 and 3, Geometry 2 was the less accurate, showing presence of partially closed pores.

SEM analysis was performed to further characterize the surface ultrastructure of PLA scaffolds; the micrographs revealed small imperfections related to the 3D printing process. Geometry 2 scaffolds were the most challenging to print and SEM analysis confirmed the presence of a higher amount of printing residues on these scaffolds.

#### In vitro HM1-SV40 cell proliferation: evaluation of scaffold-cell interaction

To investigate the interaction of the PLA scaffolds with bone marrow mesenchymal stem cells, HM1-SV40 cells proliferation after seeding was assessed after 7 and 14 days.

**Figure 18.**
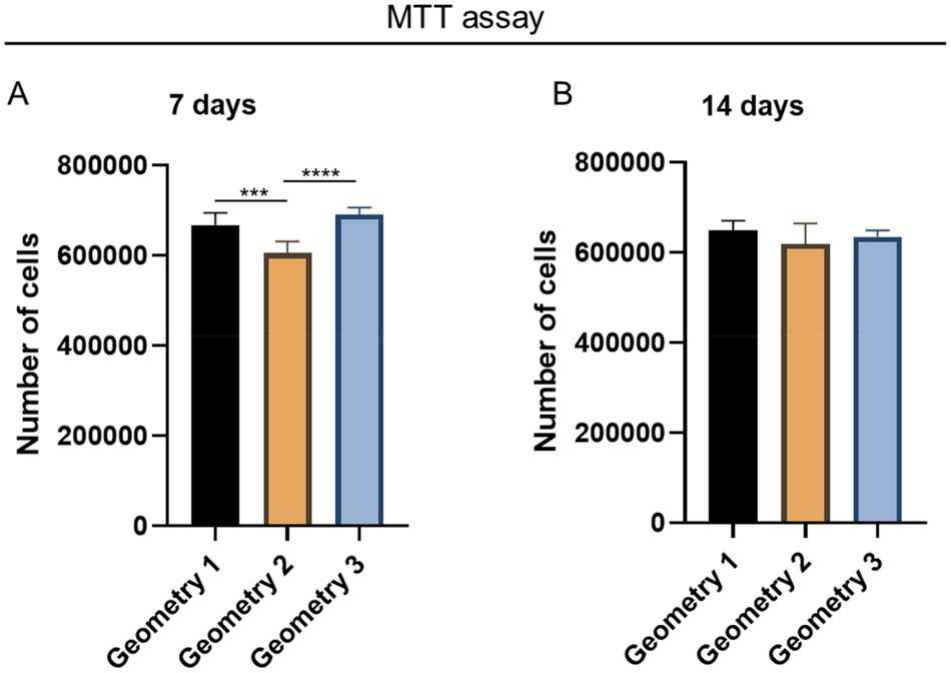
Human bone marrow mesenchymal stem cells (HM1-SV40) adhesion and proliferation on PLA scaffolds with different geometries. Cells behavior was determined by MTT assay at 7 days (**A**) and 14 days (**B**) from seeding (***P *<* 0.001; ****P *<* 0.0001).

At Day 7, all the geometries demonstrated an excellent ability to support cell adhesion and proliferation. Cell proliferation on Geometry 1 and Geometry 3 resulted significantly higher compared to Geometry 2 (*P* < 0.001 and *P* < 0.0001, respectively). After 14 days of culture, both Geometry 1 and Geometry 3 showed a slight decrease in cells number, while Geometry 2 showed a slight increase. However, no significant differences were observed among the groups (Figure 18).

**Figure 19.**
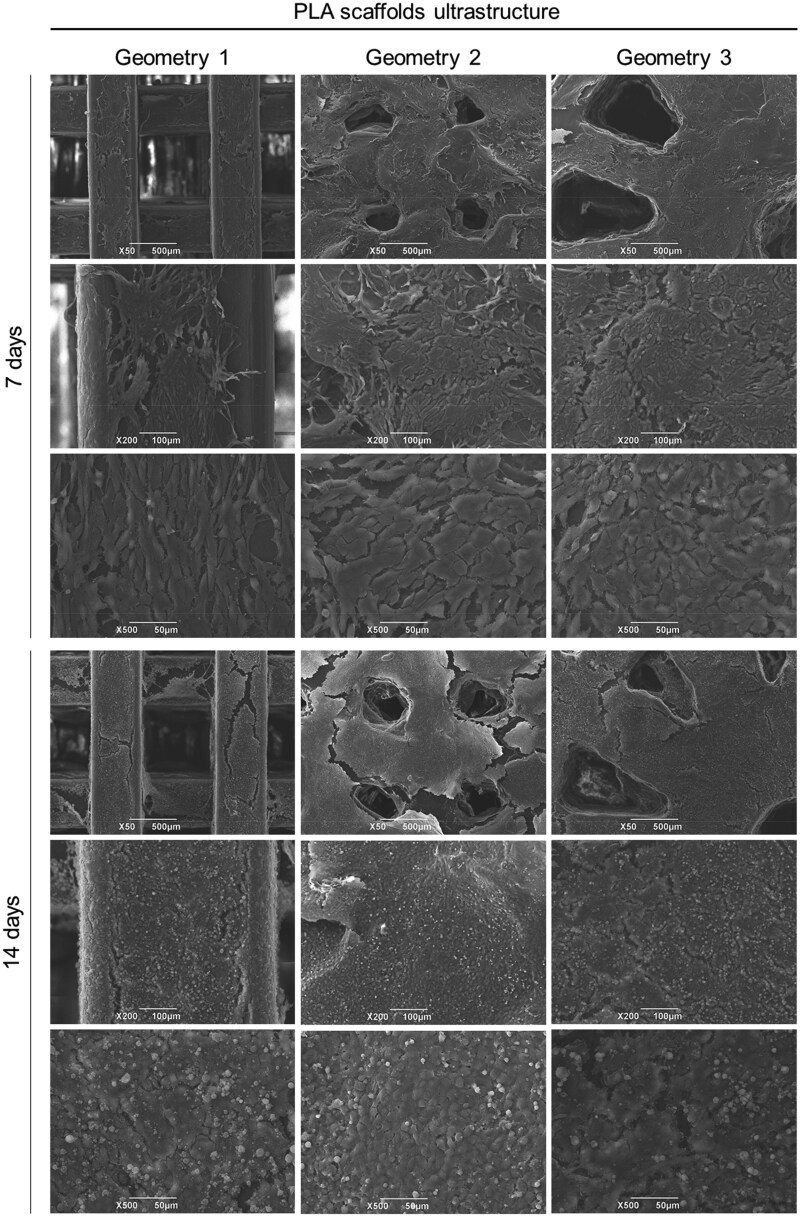
Evaluation by SEM of human bone marrow mesenchymal stem cells (HM1-SV40) adhesion and proliferation on PLA scaffolds with the three different geometries, at 7 and 14 days from seeding. Scale bars: 500 µm, 100 µm, 50 µm.

The presence of HM1-SV40 cells on PLA scaffolds was also assessed by SEM. Adherent cells were detected in all the experimental groups, at each endpoint, supporting the MTT assay results. At Day 7, cells formed a monolayer with only some spaces between them. After 14 days from seeding, no empty area was detected, some detached/roundish cell was present, indicating the reaching of full confluence because of massive proliferation. Infiltrating cells were observed also within scaffolds pores/holes (Figure 19).

## Discussion

An ideal scaffold should provide a suitable surface for cell attachment and proliferation, exhibit high biocompatibility, and possess a degradation rate that aligns with the deposition of newly formed tissue. These characteristics are essential for the success of cartilage implants and *in vivo*-associated outcomes [42]. By focusing on scaffold fabrication at the macroscale, microscale and nanoscale levels, improved cell-scaffold interactions can be achieved, which are crucial for successful tissue regeneration [43, 44]. Various techniques have been employed to create porous scaffolds for tissue engineering, with the goal of enhancing surface-to-volume ratios and mimicking the 3D ultrastructure of extracellular matrices. These strategies aim to improve cell migration, proliferation, adhesion and differentiation [45].

In this study, the promising OxPVA [46] was adopted to develop bioresorbable scaffolds for cartilage regeneration, aiming to address the polymer’s poor interaction with cells, an issue also shared with its native counterpart PVA [7, 47]. Porous OxPVA scaffolds were fabricated through physical cross-linking, using gelatin as a porogen. Specifically, gelatin microsphere leaching was employed as a method to assess how porosity parameters can influence cell proliferation [48]. To this purpose, different w/w percentages relative to the polymer weight were compared. Following the physical cross-linking of the hydrogels, gelatin was removed by soaking the scaffolds at 40°C under stirring, as described in the literature [49]. It is important to note that various methods can be used for hydrogel cross-linking, and controlling this process is vital for preparing membranes with a porous morphology [50]. For PVA, chemical cross-linking often involves aldehydes, particularly glutaraldehyde (GA) [51]; however, while GA treatment enhances scaffold resistance to water [52], physically cross-linked PVA hydrogels are often preferred in bio-related applications due to their biological safety [53]. Here, all the physically cross-linked OxPVA scaffolds maintained their stability after the porogen was removed.

Pore size is a critical factor in scaffold design, as it must fall within a range that facilitates cell penetration and migration during seeding, nutrient diffusion, metabolic waste removal, while providing for a 3D microenvironment allowing for cell assembly and potential stem cell differentiation [43, 48]. If pores are too small, cells are unable to migrate within the construct, and nutrient diffusion and waste removal are hindered. Conversely, excessively large pores can limit cell attachment [54]. In the current study, scaffolds prepared with lower gelatin concentrations (10%P w/w) showed numerous and easily identifiable superficial pores. As gelatin content increased (15%P w/w, 25%P w/w), individual pores tended to coalesce, forming progressively larger porosities (up to 79.89 ± 26.98 µm^2^ in OxPVA + 25%P vs 44.33 ± 11.56 µm^2^ in OxPVA + 10%P). A similar trend was observed in transversal sections (109.30 ± 22.11 µm^2^ in OxPVA + 25%P vs 27.00 ± 5.61 µm^2^) while pores were negligible in the control group. The presence, distribution and size of pores are important when evaluating scaffold repopulation by cells and, a 3D environment is fundamental to induce mesenchymal stem cells’ chondrogenic differentiation [55].

Bone marrow-derived stem cells are one of the preferred choices in studies evaluating the potential of scaffolds for musculoskeletal tissue engineering or regenerative medicine therapies; they can differentiate into tissues of the mesenchymal lineage, including cartilage, bone, fat and muscle and they can be isolated easily from patient’s bone marrow [56–58]. After seeding scaffolds with HM1-SV40 cells, supports with the larger pores showed to favor cell adhesion and proliferation, differently from the smooth ones. This is in accordance with Prasopthum *et al*. who demonstrated that scaffolds with micro/nanoporous structures promote chondrogenic/osteogenic differentiation of human bone marrow mesenchymal stem cells better than nonporous supports [59]. Despite stem cells differentiation was not evaluated here (this is indeed a limitation of the study), typically, the first step of bone marrow-derived stem cells chondrogenesis include mesenchymal condensation which is characterized by the formation of a compact 3D aggregate of mesenchymal stem cells. It descends that scaffolds promoting their aggregation/condensation are ideal for supporting cartilage regeneration [60]. While it is well-established that OxPVA devices, whether nonmodified or bioactivated, cannot support cell adhesion and proliferation [18, 21, 26, 61], the effects of pore size variations were not previously assessed for this material. Here, interesting results in HM1-SV40 cells adhesion/proliferation were observed with pores in the range of 79.89 ± 26.98 µm^2^; however, literature is conflicting on the optimal pore size for successful tissue engineering [54, 60]: a wide range (5–500 μm) is reported, certainly depending on the cell type [62, 63]. Scaffolds with smaller pores (125–250 μm) are more effective in promoting chondrogenic differentiation and preventing endochondral ossification of human bone marrow mesenchymal stem cells, compared to scaffolds with larger pores (425–600 μm) [60]. There are studies revealing that polycaprolactone (PCL) scaffolds with pore size of 200-μm induced a significantly higher proliferation, chondrogenic gene expression and cartilage-like ECM deposition of bone marrow mesenchymal stem cells versus scaffolds with smaller or large pore sizes (especially 50 and 400 μm) [64]. Conversely, pore size of 370–400 μm was found to provide a more favorable environment for the chondrogenic differentiation of human adipose stem cells seeded in PCL scaffolds by Oh *et al*. [65]. Other works considering gelatin supports showed that pores of 250–500 μm are better for chondrocyte proliferation and ECM secretion [66].

Porous scaffolds and natural ECMs differ in that the former lack specific biological motifs that interact with cultured cells. To address this limitation, surface functionalization strategies have been employed, incorporating ECM byproducts, proteins and peptides [47]. As previously reported, PVA- and OxPVA-based scaffolds have also been modified in this way using ECM components to meet the needs of various end-use applications [7, 18, 21]. In brief, bilayer membranes were created by integrating the mechanical properties of the polymer with the bioactivity of matrix-derived components. Both human umbilical cord Wharton’s jelly and human cartilage were decellularized, homogenized and lyophilized to produce sponges, which were then combined with the hydrogel via physical cross-linking for cartilage regeneration [7, 18].

In this study, scaffold bioactivation was also achieved through the combination of micropores (with 15%P and 25%P being preferred) and acellular cartilage suspension, which was incorporated into the hydrogel to form a blend. Cartilage acellular matrix is primarily used for cartilage defect repair and to functionalize other materials, thereby enhancing cartilage formation [67]. To optimize the desired function in terms of bioactivity, the decellularization protocol must be tissue-respectful; more than 90% of the normal cartilage tissue consists of type II collagen and GAGs [67]. Histological and biochemical analyses were performed to assess eventual variations related to tissue processing. According to study evidence, the decellularization protocol adopted here successfully preserved collagens and GAGs (with no significant difference in GAGs content), while assuring nonimmunogenicity. The bio-functionalized porous OxPVA scaffolds exhibited a homogeneous distribution of the cartilage-derived matrix suspension, further enhancing interactions with HM1-SV40 cells compared to scaffolds without the matrix suspension. Additionally, it was also possible to determine how presence of dECM can influence pores size. Compared to dECM-free supports, it was observed a general reduction of mean pores dimension along with dECM presence. These results are in accordance with Almeida *et al*. [68] who, in a comparative study, demonstrated that varying the ECM slurry concentration (250, 500 and 1000 mg ml^−1^) pore sizes were modulated. Specifically, lowering the concentration of ECM scaffolds showed a higher mean pore size (from 32 ± 12–65 ± 20 μm). Recurring to ECM homogenates and matrix macromolecules, rather than their superstructure, is undoubtedly an innovative and promising approach for scaffold functionalization [7]. Moreover, the prepared ECM suspension addressed not only the need for a dECM with retained bioactivity but also ensured it was free from additive residues that could potentially trigger negative biological responses [69]. This was further supported by *in vitro* cytocompatibility assays and *in vivo* biocompatibility studies, where only a thin fibro-connective capsule surrounding the implant was detected, consistent with the type of surgery performed [26].

In cartilage tissue engineering, evaluating the mechanical properties of a construct is essential to ensure its capacity to support functional tissue regeneration. Accordingly, in this study, in addition to assessing the biocompatibility and bioactivity of porous OxPVA hydrogels, with or without dECM, we investigated their biomechanical performance. Consistently with previous findings [38], nonporous OxPVA exhibited a compressive response comparable to the lower range of human articular cartilage stiffness (approximately 200–5400 kPa [70]). In porous hydrogels, the presence of void spaces, corresponding to porosity levels of approximately 10%, 15% and 25%, reduced the solid fraction of the scaffold and, consequently, its ability to resist compressive deformation. Both the purely porous hydrogels (OxPVA + 10%P, OxPVA + 15%P, OxPVA + 25%P) and the porous scaffolds enriched with dECM (OxPVA + 10%P+dECM, OxPVA + 15%P+dECM, OxPVA + 25%P+dECM) exhibited reductions in compressive stiffness of up to one third with respect to the bulk material. Since porosity is essential for enhancing scaffold bioactivity despite its negative effect of scaffold structural stiffness, in future studies, the stiffness reduction associated with it, could be mitigated by applying additional crosslinking strategies, such as irradiation or chemical cross-linking [71], to strengthen the polymer network and improve mechanical performance.

With respect to viscoelastic behavior, bulk OxPVA displayed a long-term force reduction of approximately 40%, as observed in previous studies [38]. This time-dependent mechanical response differs from that of native articular cartilage, which exhibits a force reduction of around 60% under compressive deformation [72]. The introduction of porosity increased the equilibrium force decay, resulting in values closer to those of native cartilage. Previous studies have reported that the impact of ECM incorporation on the mechanical properties of natural or synthetic polymers depends on parameters such as ECM content, form (solubilized, particulate or coating), polymer type and crystallinity and fabrication method [73, 74]. In the present work, incorporating dECM into the OxPVA matrix did not alter the instantaneous compressive response, with stiffness values remaining around 150 kPa and only slightly influenced the viscoelastic behavior. Similarly, Kim *et al*. [75] reported that adding ECM components to PVA hydrogels did not measurably change bulk mechanical properties in the absence of cellular activity, with significant reinforcement occurring only after cell-mediated ECM deposition within the scaffold network. The modest change in viscoelastic behavior observed here may be attributed to the hydrophilic components of the dECM, which could influence fluid mobility within the polymer matrix under constant strain.

A scaffold for cartilage tissue engineering should maintain homogeneous cell viability during construct development by presenting a well-interconnected pore network that supports nutrient and oxygen transport, facilitates waste removal and enables the release of bioactive factors such as proteins and cytokines [76]. To evaluate these transport-related properties, we performed FRAP analysis on both porous OxPVA and porous OxPVA+dECM hydrogels, measuring the half-recovery time (t_1_/_2_), which inversely correlates with diffusivity [77, 78]. Specifically, we assessed the diffusion of 500 kDa dextran as a model for physiologically relevant macromolecules, allowing us to extrapolate the scaffold’s ability to transport a range of solutes, from small molecules such as amino acids (∼100 Da) and monosaccharides (∼200 Da) to large proteins and growth factors of several hundred kilodaltons.

Our results demonstrated that increasing scaffold porosity to 10% and 15%, decreased *τ*_1/2_, indicating enhanced molecular transport through the scaffold. This finding aligns with the work of Offeddu *et al*. [79], who reported that larger pores reduce resistance to solute movement and increase fluid permeability, thereby facilitating diffusion. However, further increasing porosity to 25% reversed this trend, with *τ*_1/2_values rising. This suggests that transport efficiency does not depend solely on pore size; at high porosity levels, a reduction in interconnectivity may limit the number of continuous diffusion pathways. Incorporation of dECM into the porous polymer did not significantly affect diffusivity, indicating that dECM components did not obstruct molecular transport within the scaffold network.

Chondrogenesis is a highly coordinated event involving several hormones, growth factors, cytokines and transcription factors [80]. The results obtained from the gene expression analysis suggest that the tested bioactive supports can modulate cellular activity over time, promoting the expression of key genes involved in cartilage formation and tissue remodeling. In particular, the increased mRNA levels of COL2A1, which encodes the main type II collagen component of cartilage [81], indicate that the OxPVA supports combined with ECM can effectively support chondrogenic differentiation. The observed upregulation (up to 1.5-fold) is particularly significant, as type II collagen is a hallmark marker of functional hyaline cartilage.

Although the increases in COL9A1 and COL10A1 were less pronounced, their expression supports the idea that these materials also contribute to the later stages of chondrogenesis and cartilage maturation. COL10A1 is typically associated with chondrocyte hypertrophy [82, 83], suggesting the scaffolds promote not only early differentiation but also progression toward a mature cartilage phenotype.

The increase of COMP after 14 days in both OxPVA 25%P and OxPVA 25%P+ECM further confirms the effectiveness of these materials in promoting the synthesis of cartilage-specific ECM components. COMP is considered an early marker of chondrogenic differentiation and it contributes to matrix formation, stabilization, organization [84] and improved cartilage mechanical properties [85].

SOX genes’ expression, which typically occurs early in chondrogenesis [86], was also focused. SOX9 is a key transcription factor with a role in initiating and regulating chondrogenesis; it is expressed by mesenchymal stem cells (MSCs) during the early condensation and it controls the expression of cartilage-specific genes independently or in cooperation with SOX5 and SOX6 (together forming the chondrogenic SOX trio) [80]. Typicallly, SOX9 remains upregulated during chondroprogenitor proliferation and differentiation, playing a crucial role in both initiating chondrogenesis and maintaining the chondrocyte phenotype [87]. In this study, SOX9 expression progressively increased over 14 days, with the highest levels detected in ECM-enriched scaffolds, highlighting ECM components’ role in providing critical biochemical cues to sustain chondrogenic commitment. SOX5 also plays a significant role in promoting chondrogenesis [80, 88]. In this study, SOX5 expression steadily increased over time, regardless of the support used, indicating a temporally regulated but material-independent role during chondrogenic progression [88]. The sustained upregulation of both transcription factors supports SOX 9 and SOX5 coordinated involvement across multiple stages of chondrogenesis, from early lineage specification to ECM production/stabilization.

In line with the need for collagen biosynthesis and stabilization, the time-dependent increase in P4HA1, which encodes an enzyme involved in the hydroxylation of proline residues during collagen synthesis [89, 90], highlights the active role of ECM-enriched supports in promoting the formation of a well-organized and functionally competent ECM.

Finally, the activation of MMP14 and MMP16, two matrix metalloproteinases involved in matrix degradation and remodeling [91], suggests that the bioactive supports not only promote cartilage matrix deposition but also support the dynamic remodeling processes necessary for proper tissue organization/adaptation. The absence of a clear time-dependent association in their expression may suggest a constitutive regulation influenced more by scaffold bioactivity and/or ultrastructure than by culture time.

Overall, study results related to expression of genes related to collagen formation, cartilage differentiation and remodelling confirm that OxPVA supports, particularly the 25%P enriched with ECM, can likely provide a favorable environment supporting chondrogenic differentiation and functional cartilage matrix deposition. The concerted activation of genes involved in key processes such as collagen synthesis, chondrogenic regulation and tissue remodeling highlights the potential of porous OxPVA scaffolds (especially when combined with ECM) as promising candidates for cartilage tissue engineering applications.

Integrating qPCR results, protein expression analysis provided further insights into the chondrogenic potential of the different scaffold formulations with respect to porosity and dECM incorporation. The significant increase in SOX9 expression after dECM addition into OxPVA + 15%P scaffolds suggests that the bioactive cues provided by the cartilage matrix components may guide the early commitment of MSCs toward the chondrogenic lineage. Similarly, ACAN expression tended to increase following the incorporation of dECM within the synthetic scaffolds, although this effect did not reach statistical significance in both porosity configurations. Considering that ACAN is one of the main structural proteoglycans of cartilage ECM, this result may indicate an early stimulation of matrix deposition, which might become more evident at longer culture time points [92]. The expression of COMP appeared to be mainly influenced by scaffold porosity, with higher levels detected in the OxPVA25%P groups regardless of dECM incorporation. This finding suggests that the increased pore size and interconnectivity may favor cell infiltration and three-dimensional organization, thereby promoting the synthesis of COMP, which plays a master role in cartilage matrix assembly and structural integrity [93].

PLA is a renewable, biodegradable and biocompatible polymer, making it an ideal material for biomedical applications, particularly in the production of implants for bone tissue engineering, including 3D-printed scaffolds [94, 95]. In this context, rapid prototyping is a valuable tool for fabricating supports on demand, enabling the creation of *in vitro* platforms for the evaluation of the effects of various parameters on cell behavior [96]. One of the key advantages of this technique is its ability to produce structures with interlocking pores, featuring highly precise and controllable sizes and geometries [97, 98]. Diomede *et al*. [99] demonstrated that mesenchymal stem cells seeded onto 3D-PLA can induce a regenerative bone process without immunogenic effects *in vivo*. Pores size/characteristics, total porosity and fiber width of PLA scaffolds are critical factors in promoting proper cell adhesion and ensuring a suitable PLA degradation rate for tissue scaffolding [100, 101]. For effective bone tissue regeneration, scaffolds must fulfill several key requirements: they must have interconnected pores to allow for cell diffusion and migration, provide sufficient surface area for interaction with surrounding tissues, and maintain mechanical stability without being weakened by the porous structure. A minimum pore size of approximately 100 µm is needed to support cell ingrowth (pore size < 100 µm is associated with the formation of nonmineralized osteoid or fibrous tissue [102]); whereas, pores larger than 300 µm are recommended to improve vascularization and enhance osteogenesis [102–104], with overall porosity ranging from 50% to 90% (recommended porosity is 90% [105–107]. The scaffolds developed in this study (geometries 1–3) were designed following these criteria but, in the perspective to avoid eventual mechanical properties issues possibly associated with high porosity, we compared lower values as previously suggested by Gregor *et al*. [107]. Regarding fiber width, it is suggested to fall within a range between 200 and 900 μm [108]. In consideration of that, a range from 500 to 650 μm was preferred. After HM1-SV40 cells seeding, all three PLA geometries proved to be adequate in promoting cell adhesion; initial differences in proliferation at Day 7 were overcome after 14 days. Comparing the two end-points no variation was detected in terms of cells proliferation and a plateau phase can be assumed to be reached.

Overall, the study results highlight the potential of porous OxPVA + dECM and 3D-printed PLA scaffolds in promoting mesenchymal stem cell adhesion and proliferation. For the first time, the introduction of a specific degree of porosity has been shown to effectively enable OxPVA cellular colonization, which would otherwise not be possible. This effect is further enhanced by the presence of dECM, which facilitates the bioactivation of the synthetic polymer. In the case of PLA supports, the results suggest that a balance between pore size and porosity percentage can be achieved without compromising interactions with cells. This finding is particularly significant for future *in vivo* implantation, where scaffolds are expected to endure mechanical loading.

## Conclusions

The treatment of osteochondral lesions remains a significant medical challenge. When the osteochondral unit is damaged, it does not heal spontaneously, which increases the risk of developing long-term degenerative joint diseases such as OA [109]. Consequently, extensive research into the design/fabrication of scaffolds using tissue engineering strategies has emerged, with the aim of effectively address and manage these defects [110].

Here, porous OxPVA-based scaffolds eventually combined with dECM were developed and characterized. Increased porosity significantly influenced the microstructure, mechanical properties and bioactivity of the scaffolds. Scaffolds fabricated with higher porogen content and dECM showed an improved cell proliferation associated with upregulated expression of cartilage-specific genes. Mechanical analyses showed a decrease in stiffness along with increased porosity, while viscoelastic properties remained stable with dECM addition. *In vivo* biocompatibility study confirmed the absence of severe inflammatory responses following subcutaneous implantation. Overall, these findings prove the potential of porous OxPVA + dECM composite scaffolds as effective biomaterials for osteochondral tissue engineering. Future research should focus on optimizing scaffold mechanical strength, enhancing stem cell differentiation and performing long-term *in vivo* studies to confirm clinical efficacy. Additionally, varying dECM concentrations should be investigated both *in vitro* and *in vivo*.

PLA scaffolds with different geometries were also produced and proved their ability in supporting HM1-SV40 proliferation. Bone tissue has an intrinsic self-healing capacity; however, in cases of severe damage, it is possible to enhance regeneration by recurring to auxiliary treatments. While cellular constructs alone are effective in bone tissue engineering, noncellular approaches can also yield promising results [111]. Biocomposite scaffolds, made of polymers and bioactive materials, represent an intriguing strategy for improving both mechanical properties and bioactivity [112, 113]. The supports must stimulate cells migration/growth once implanted at the defect site and are expected to be replaced by host cells in turn secreting and depositing tissue-specific ECM, in turn leading to native-like tissue [114, 98]. In this context, although PLA is a promising material for bone tissue engineering, its mechanical strength and osteoconductive properties still require optimization. Conjugating osteoinductive biomolecules to PLA scaffolds presents a valuable opportunity for further functional enhancement.

## Supplementary Material

rbaf109_Supplementary_Data

## Data Availability

The datasets used and/or analysed during the current study are available from the corresponding author on reasonable request.
