# Supplementary material for: The impact of microstructure and extracellular matrix suspension on the proliferation of bone marrow-derived mesenchymal stem cells for osteochondral defect repair
Source: Regen Biomater. 2025 Nov 12;12:rbaf109. doi: 10.1093/rb/rbaf109 (PMC12718104; doi:10.1093/rb/rbaf109)
Supplement: rbaf109_Supplementary_Data [file rbaf109_supplementary_data.pdf]

## Supplementary Table 1

**Table 1.** Gene expression assayed by quantitative PCR

| | Fold change *vs* ctrl | | | | | | | |
|---|---|---|---|---|---|---|---|---|
| | **OxPVA 15%P** | | **OxPVA 25%P** | | **OxPVA 15%P+ECM** | | **OxPVA 25%P+ECM** | |
| **gene** | **7 days** | **14 days** | **7 days** | **14 days** | **7 days** | **14 days** | **7 days** | **14 days** |
| ***Formation*** | | | | | | | | |
| ***COL2A1*** | 2.53±0.38 | 2.43±0.34 | 5.31±0.32 | 7.12±0.31 | 4.51±0.28 | 6.75±0.43 | 7.74±0.27 | 9.76±0.09 |
| ***COL9A1*** | 1.52±0.13 | 2.07±0.17 | 3.27±0.13 | 4.33±0.17 | 3.29±0.30 | 4.08±0.32 | 5.18±0.26 | 6.23±0.06 |
| ***COL10A1*** | 1.05±0.09 | 1.25±0.03 | 1.33±0.07 | 1.54±0.07 | 1.58±0.11 | 1.83±0.14 | 2.14±0.08 | 2.16±0.35 |
| ***COMP*** | 3.79±0.62 | 3.57±0.73 | 8.37±0.53 | 11.35±0.52 | 7.05±0.46 | 10.75±0.71 | 12.38±0.45 | 15.67±0.67 |
| ***Differentiation*** | | | | | | | | |
| ***SOX5*** | 1.02±0.10 | 2.04±0.06 | 1.26±0.09 | 2.41±0.12 | 1.27±0.07 | 1.99±0.09 | 1.37±0.06 | 2.03±0.25 |
| ***SOX9*** | 1.27±0.12 | 1.73±0.05 | 1.63±0.13 | 2.21±0.11 | 1.96±0.04 | 2.98±0.13 | 2.40±0.06 | 4.68±0.51 |
| ***BMP2*** | 2.24±0.32 | 2.94±0.30 | 3.65±0.22 | 5.35±0.20 | 4.03±0.15 | 6.45±0.40 | 4.98±0.14 | 7.23±0.20 |
| ***BMP6*** | 1.57±0.22 | 2.16±0.29 | 1.59±0.12 | 2.08±0.19 | 1.82±0.10 | 2.18±0.26 | 2.15±0.26 | 2.72±0.41 |
| ***P4HA1*** | 1.55±0.12 | 3.75±0.23 | 1.45±0.07 | 4.52±0.08 | 1.82±0.13 | 6.45±0.30 | 2.27±0.14 | 8.32±0.20 |
| ***Remodelling*** | | | | | | | | |
| ***MMP14*** | 1.23±0.10 | 1.88±0.13 | 2.09±0.10 | 2.16±0.16 | 2.84±0.32 | 3.22±0.25 | 2.98±0.22 | 3.20±0.05 |
| ***MMP16*** | 1.69±0.10 | 2.48±0.17 | 2.72±0.11 | 2.84±0.22 | 3.32±0.22 | 4.41±0.24 | 3.25±0.22 | 4.55±0.13 |
